# Expansion of murine and human olfactory epithelium/mucosa colonies and generation of mature olfactory sensory neurons under chemically defined conditions

**DOI:** 10.7150/thno.46750

**Published:** 2021-01-01

**Authors:** Wenwen Ren, Li Wang, Xiujuan Zhang, Xiaoyu Feng, Liujing Zhuang, Nan Jiang, Rui Xu, Xuewen Li, Ping Wang, Xicai Sun, Hongmeng Yu, Yiqun Yu

**Affiliations:** 1 Department of Otolaryngology, Eye, Ear, Nose and Throat Hospital, Shanghai Key Clinical Disciplines of Otorhinolaryngology, Fudan University, Shanghai, China 200031.; 2 School of Life Sciences, Shanghai University, Shanghai, China 200444.; 3 Biosensor National Special Laboratory, Key Laboratory for Biomedical Engineering of Education Ministry, Department of Biomedical Engineering, Zhejiang University, Hangzhou, China 310027.; 4 Institutes of Biomedical Sciences, Fudan University, Shanghai, China 200031.

**Keywords:** Olfactory epithelium/mucosa, three-dimensional culture, colony, Lgr5, olfactory sensory neurons

## Abstract

Olfactory dysfunctions, including hyposmia and anosmia, affect ~100 million people around the world and the underlying causes are not fully understood. Degeneration of olfactory sensory neurons and incapacity of globose basal cells to generate olfactory sensory neurons are found in elder people and patients with smell disorders. Thus, olfactory stem cell may function as a promising tool to replace inactivated globose basal cells and to generate sensory neurons.

**Methods:** We established clonal expansion of cells from the murine olfactory epithelium as well as colony growth from human olfactory mucosa using Matrigel-based three-dimensional system. These colonies were characterized by immunostaining against olfactory epithelium cellular markers and by calcium imaging of responses to odors. Chemical addition was optimized to promote Lgr5 expression, colony growth and sensory neuron generation, tested by quantitative PCR and immunostaining against progenitor and neuronal markers. The differential transcriptomes in multiple signaling pathways between colonies under different base media and chemical cocktails were determined by RNA-Seq.

**Results:** In defined culture media, we found that VPA and CHIR99021 induced the highest Lgr5 expression level, while LY411575 resulted in the most abundant yield of OMP^+^ mature sensory neurons in murine colonies. Different base culture media with drug cocktails led to apparent morphological alteration from filled to cystic appearance, accompanied with massive transcriptional changes in multiple signaling pathways. Generation of sensory neurons in human colonies was affected through TGF-β signaling, while Lgr5 expression and cell proliferation was regulated by VPA.

**Conclusion:** Our findings suggest that targeting expansion of olfactory epithelium/mucosa colonies *in vitro* potentially results in discovery of new source to cell replacement-based therapy against smell loss.

## Introduction

The National Institute on Deafness and Other Communication Disorders (NIDCD) estimates that ~1.4% of the United States population experience chronic olfactory dysfunction and smell loss 1. Olfactory dysfunction results from several etiologies, the majority of which include sinonasal disease, head trauma, upper respiratory infection, age-related loss and congenital disorders 2. Sense of smell depends on the mature sensory neurons expressing olfactory receptors (ORs), located on the olfactory epithelium (OE) 3. The basal cells in the OE are able to generate sensory neurons when the epithelium is injured 4. However, the regenerative capacity of basal cells is not limitless 5. For instance, the OE from elderly humans contains areas that lack of sensory neurons and globose basal cells (GBCs) 6. Meanwhile, nasal diseases such as chronic rhinosinusitis cause neuroepithelial reorganization in animal models 7 while inflammation, a typical indicator to chronic rhinosinusitis, attenuates the regenerative capacity of olfactory stem cells 8. Thus, olfactory basal/stem cells are potentially vulnerable to aging and disease progression, resulting in smell loss and other disorders due to incapacity of sensory neuron regeneration 9.

Two subtypes of basal cells, horizontal basal cells (HBCs) and GBCs, are responsible for OE regeneration 4. HBCs are dormant reserve stem cells that respond to severe epithelial injury 10, 11 while heterogeneous GBCs are proliferative and able to generate sensory neurons in the OE 12. GBCs express Leucine-rich repeat-containing G-protein coupled receptor 5 (Lgr5) 13, 14, a bona fide biomarker for stem cells in multiple tissues 15, playing roles in tissue regeneration and development 16, 17. Since Lgr5^+^ cells generate multiple cell lineages in the OE after methimazole-caused injury 13, they may play significant roles in regeneration of sensory neurons as well as OE repair from injury.

Olfactory stem cells have been established *in vitro* as cloned neurospheres from rat 18, mouse 19 and human 20. Promisingly, these cells are optimal candidates to the source of cell transplantation in tissue repairs including facial nerve regeneration 21, restoration of normal substantia nigra structure 22, improvement of stroke-mediated neurological dysfunction 23, restoration of motor functions in spinal cord injury model 24, recovery of neuroplasticity in hippocampal lesions 25 and preservation of auditory function during early-onset progressive hearing loss 26. Meanwhile, transdifferentiation into dopaminergic neurons suggests that olfactory stem cells may function as a promising model for drug screen and optimal source for Parkinson's disease treatment 27, 28. Therefore, simple but efficient method such as using small molecule chemicals and growth factors to maintain stemness and induce differentiation of olfactory stem cells is worth further exploration and validation.

In this study, we cultured Lgr5^+^ cells sorted from the murine OE *in vitro* using a Matrigel-based three-dimensional system. Colonies derived from adult Lgr5^+^ stem cells have been established *in vitro*, including cells from the tongue 29, cochlea 30, small intestine 31, and liver 32. Proliferation and differentiation of colonies derived from Lgr5^+^ cells were regulated under different growth conditions 30, 31, 33. Here, we defined chemicals and growth factors regulating Lgr5 expression in cultured colonies from the murine OE. We reported the culture condition generating the highest yield of OMP^+^ olfactory sensory neurons in OE colonies. Morphological change from filled to cystic colony was observed in different base media with cocktail treatments, which was associated with differential Lgr5 expression and transcriptional alteration in multiple signaling pathways. Furthermore, we also established colonies cultured from human olfactory mucosa, and colony proliferation and neuronal differentiation were regulated by defined chemical stimulations. Thus, this study put forward a new source for cell-based transplantation therapy against smell loss.

## Materials and Methods

### Animals

Genetically targeted heterozygous Lgr5-EGFP-IRES-CreERT2 mice (Stock number 008875, harboring a "knock-in" allele that abolishes Lgr5 gene function and expresses EGFP and CreERT2 fusion protein from the Lgr5 promoter/enhancer elements), Rosa26-floxed STOP-TdTomato mice (Stock number 007909, a Cre reporter strain with a LoxP-flanked STOP cassette prevents transcription of the downstream red fluorescent protein) and OMP-Cre mice (Stock number 006668, the coding region and part of the 3' non-translated region of the OMP gene was replaced by Cre) were purchased from the Jackson Laboratory. Both male and female mice were used in this study and the data were grouped together because no sex difference was evident. The procedures of animal handling and tissue harvesting were approved by the Shanghai Medical Experimental Animal Administrative Committee (Permit Number: 2009-0082).

### Chemicals

All reagents were purchased from Sigma Aldrich unless specified. Chemicals were prepared as stock solutions and diluted freshly before using. The final concentration of each chemical was: 50 ng/mL bFGF (basic fibroblast growth factor, ThermoFisher, # 13256029), 50 ng/mL EGF (epidermal growth factor, ThermoFisher, #PHG0311), 50 ng/mL IGF-1 (insulin-like growth factor 1, STEMCELL Technologies, #78022), 3 μM CHIR99021 (#SML1046), 1 mM VPA (Valproic acid, #P4543), 100 μg/mL pVc (L-ascorbic acid 2-phosphate, #49752), 2 μM 616452 (Calbiochem, #446859-33-2), 10 mM Nicotinamide (#N0636), 12.5 mM N-Acetyl-L-cysteine (#A9165), 10 μM SB431542 (#S4317), 5 μM LY411575 (#SML0506), 10 μM SB202190 (#S7067), 100 ng/mL SHH (Sonic hedgehog, #SRP3156) and 500 nM A83-01 (#SML0788).

### Cell sorting

To obtain Lgr5^+^ cells, the OEs from Lgr5-EGFP-IRES-CreERT2 mice (both male and female) at 3-month-old age were minced into small pieces using scissors in Tyrode's solution (145 mM NaCl, 5 mM KCl, 10 mM HEPES, 5 mM NaHCO_3_, 10 mM pyruvate, 10 mM glucose), then treated with 0.25% Trypsin-EDTA (ThermoFisher, #25200056) and DNase I (final concentration of 40 U/mL, Sigma-Aldrich, #D5025) for 20-30 min at 37 °C. Cell suspension was collected by centrifugation at 200 g. Cell pellet was suspended in HBSS buffer (HBSS containing 5 mM MgCl_2_, 10 mM HEPES, 50 μg/mL DNase I, 0.1 g/mL bovine serum albumin, 10 μg/mL DAPI and 5% fetal bovine serum) using a fire-polished glass pipette. Single-cell suspension was filtered using 70-μm nylon mesh (BD Falcon, #352350) to remove large aggregates, followed by further filtering with 35-μm nylon mesh (BD Falcon, #352235). Cells were sorted using BD FACS Aria III cell sorter (BD Biosciences), according to the green fluorescent EGFP signal (excitation, 488 nm; emission, 530 nm). Cells were sorted into a low-attached 1.5-mL microcentrifuge tube containing 0.5 mL colony growth medium described below.

### Colony culture from murine olfactory epithelium

Sorted Lgr5^+^ cells from the OE were cultured according to previously reported protocol with some modifications 14. Sorted cells were seeded in low-attached 24-well plate (Corning, #3473) at a density of ~ 10000 cells per well. Green fluorescence was checked by an inverted microscope (Leica DMi8). The growth medium was based on DMEM/F12 medium (ThermoFisher, #10565018) supplemented with R-spondin-1 (200 ng/mL, R&D, #4645-RS), Noggin (100 ng/mL, PeproTech, #250-38), Wnt3a (50 ng/mL, R&D, #5036-WN-010), Y27632 (10 μM, Sigma Aldrich, #Y0503), N2 (1%, ThermoFisher, #17502001), B27 (2%, ThermoFisher, #17504044), GlutaMAX™ Supplement (Thermofisher, #35050061), HEPES (10 mM, Thermofisher, #15630080) and Matrigel [4% (Vol/Vol), BD Biosciences, #356231]. Medium was changed every 3-5 days. Colonies were visible on Day 3 after* in vitro* culture and passaged every 7-10 days by using 0.25% Trypsin-EDTA to make single cell suspension. Approximately 5000 cells were reseeded in each well of low-attached 24-well plate.

### Colony culture from human olfactory mucosa

The written consents were obtained from patients before experiment. The procedures of human tissue harvesting and handling were approved by the Ethics Committee of Eye, Ear, Nose & Throat Hospital, Fudan University (Permit Number: 2019081). Human olfactory mucosae were dissected from patients with olfactory neuroblastoma. When tumors were resected, connected olfactory mucosae were isolated from tumor tissues and kept in iced PBS. Blood cells were removed by incubation with Red Blood Cell Lysis Solution (Miltenyi, #130-094-183). Tissues were minced into small pieces and digested with 0.25% Trypsin-EDTA and 50 μg/mL DNase I. Cell pellets were collected in growth medium that was used in murine OE colony culture without addition of Matrigel and then filtered with 35-μm nylon mesh (BD Falcon). Cell suspension was resuspended in growth medium supplemented with 100 μg/mL Primocin^TM^ (InvivoGen, #ant-pm-1), 2 μM 616452, 500 nM A83-01 and/or 10 μM SB431542. Approximately 10000 single cells were seeded per well in low-attached 24-well plate. Colonies were visible on Day 7 and then were passaged every 10 days or based on colony growth status.

### Viral infection

The shLgr5-mCherry lentivirus targeting the mouse Lgr5 and shCtrl-mCherry control lentivirus were prepared by GENECHEM (Shanghai, China). Viral infection in murine OE colonies was conducted when passaging. Approximately 1000 and 5000 single cells were seeded into each well of low-attached 96- and 24-well plates, infected with 0.5×10^5^ and 2.5×10^5^ TU lentivirus, respectively (MOI = 50). Apparent red fluorescence was observed on Day 3 post infection and colonies were analyzed on Day 10.

### Lineage tracing in murine OE colonies

For lineage tracing of Lgr5^+^ cells in OE colonies derived from Lgr5-CreERT2/Rosa26-TdTomato mice, 4-Hydroxytamoxifen (Sigma Aldrich, #H6278) at final concentration of 400 ng/mL was added into cultures on Day 0 post passaging and kept for 7 days. Colonies were subjected to further analysis on Day 10. Chemicals including small molecules and growth factors were added on Day 0 post passaging. A scheme describing the lineage tracing protocol was shown as Figure S3A.

### Neuronal differentiation in OE colonies

The differentiation medium was based on Neurobasal^TM^-A medium (ThermoFisher, # 10888022) containing N2 (1%), B27 (2%), GlutaMAX™ Supplement, HEPES (10 mM) and Matrigel [3% (Vol/Vol)]. To induce neuronal differentiation, Neurobasal^TM^-A differentiation medium was added on Day 10 post* in vitro* culture. LY411575 and/or CHIR99021 was added on Day 11 and colonies were incubated with these chemicals for 7 days. The scheme for differentiation protocol was shown as Figure 4A.

### Treatment with EFI-based medium

The growth medium [known as NWR (Noggin/Wnt3a/R-Spondin 1)-based medium] was replaced by DMEM/F12 medium containing B27, N2, GlutaMAX™ Supplement, HEPES and 3% Matrigel, supplemented with 50 ng/mL EGF, 50 ng/mL bFGF and 50 ng/mL IGF-1 (known as EFI-based medium). The EFI-based medium was added on Day 4 post colony passaging.

### Cryosection preparation

The protocol was described previously 34. Briefly, colonies were collected through centrifuging, washed with PBS, fixed in 4% paraformaldehyde for 15 min, and dehydrated in 30% sucrose at 4 °C overnight, followed by being wrapped into warm gelatin/sucrose solution and placed at 37 °C for 15 min to equilibrate the cells. Then, colonies in small amount of gelatin/sucrose solution (~ 20 μL) were solidified at room temperature and then were covered completely by warm gelatin/sucrose solution, which was polymerized at 4 °C. The entire block of gelatin was immersed in cold bath. The sections were prepared using a Cryostat (Leica CM1950).

### Immunohistochemistry

For the immunostaining on cultured colonies, the cryosection (prepared as described above) were washed by PBS, followed by incubation with SuperBlock^TM^ blocking buffer (ThermoFisher, #37535) containing 2% (Vol/Vol) donkey serum and 0.3% Triton X-100 at room temperature for 1 h. Primary antibody incubation was performed overnight at 4 °C. After washing three times with PBS, appropriate secondary antibodies were used to visualize staining. The nuclei were counterstained with DAPI (ThermoFisher, #62248). Slides were mounted in Vectashield Antifade Mounting Medium (Vector Laboratories, # H-1000). The primary antibodies used in this study were as follows: chicken anti-OMP (1:500, provided by Dr. Qizhi Gong at UC Davis) 35, mouse anti-Tuj1 (1:200, Abcam, #ab78078), mouse anti-Sus4 (1:200, provided by Dr. James Schwob at Tufts University) 36, rabbit anti-GFP (1:500, ThermoFisher, #A11122), mouse anti-Mash1 (1:100, R&D, #AF2567), mouse anti-Sox2 (1:100, Santa Cruz, #sc-365823), mouse anti-Ki67 (1:100, BD Biosciences, # 550609), rabbit anti-Gα_olf_ (1:100, Santa Cruz Biotechnology, #sc-55545), rabbit anti-mOREG/MOR174-9 (1:300, Novus Biologicals, #R-153-100) and rabbit anti-PGP9.5 (1:200, Proteintech, #14730-1-AP). Fluorescent images were captured under a SP8/Leica confocal microscope with LAS AF Lite software. Contrast and brightness of images were set in an equal level when captured. All captured images were z optical sections.

### Quantitative real time PCR

Total RNA was extracted by using E.Z.N.A.® Total RNA Kit I (Omega, #R6834-02) according to the manufacturer's manual. The extracted RNA was immediately dissolved in RNase-free water, and purity and concentration were determined on Biophotometer (Metash, Shanghai, China). First-strand cDNA was synthesized using a PrimeScript™ RT Master Mix (Takara, #RR036A). Primers used in this study were synthesized by Ruidibio (Shanghai, China). Quantitative real time PCR was performed on an Analytikjena Real-Time PCR System (Jena, Germany). The reaction mixtures included cDNA template, 0.2 mM primers, SYBR qPCR SuperMix (Novoprotein, #E096-01B) and ddH_2_O. Reaction conditions included an initial denaturation at 95 °C for 1 min, followed by 40 cycles of 95 °C for 20 s, 60 °C for 20 s and 72 °C for 30 s. The relative expression level was calculated using the 2^-ΔΔCt^ method. Primers sequences were as follows: mouse Lgr5: CCTGTGGCTAGATGACAATGCTCTC and AAGGCGTAGTCTGCTATGTGGTGTA. Human Lgr5: ACCTATCGTCCAACCTCC TGTCGTC and GCACAGCACTGGTAAGCATAAGGCA. Human OMP: AGTCTGT GTACCGCCTCAACTTCA and TCTATGGCATCCGAGTCCTCCTTG. Human Krt5: GAGATCGCCACTTACCGCAAGC and CATAGCCACTGCCACTGCCATAT C. Human GAPDH: GGAGCGAGATCCCTCCAAAAT and GGCTGTTGTCATACT TCTCATGG. Mouse GAPDH: TCAATGAAGGGGTCGTTGAT and CGTCCCGTAG ACAAAATGGT.

### Western blot

Proteins were extracted using a Total Protein Extraction Kit (Comiike, #PE1202). Samples were prepared by boiling in 5×SDS Loading Buffer for 5 min. SDS-PAGE was used to separate total protein, and 3 μg sample was loaded into each lane. The proteins were transferred to PVDF membrane and the membrane was blocked in 5% non-fat milk for 1 h. The following primary antibodies were used to detect protein expression: Lgr5 (Biosciences, 1:500, #LS-C98619) and β-actin (Abcam, 1:2000, #ab8226). After incubation in primary antibodies at 4 °C overnight, membrane was incubated with secondary HRP antibodies at room temperature for 1 h. Protein bands were visualized using ECL Substrate (ThermoFisher, #35050).

### RNA-Seq

RNA-Seq analysis was conducted by Majorbio Corp. (Shanghai, China). Sequencing reads were mapped to the mouse genome using HISAT2. Transcriptomes from RNA-seq reads were reconstructed by StringTie. Expression differences were evaluated using DESeq2. Pearson's coefficient was calculated to determine the correlation among different groups. The clustering analysis of global gene expression pattern in different samples was carried out using K-means clustering algorithm by RSEM software. Gene Ontology (GO, http://www.geneontology.org/) and Kyoto Encyclopedia of Genes and Genomes (KEGG) pathway analysis (http://www.genome.jp/kegg/) were performed. All the sequence data was analyzed on I-Sanger (www.i-sanger.com).

### Calcium imaging

OE colonies on Day 14 post *in vitro* culture were collected and reseeded onto the confocal dish. Then colonies were cultured in NWR-based medium for 24 h. For calcium dye loading, colonies were incubated with 5 μM fluorescent Ca^2+^ indicator Fluo-4 AM (ThermoFisher, F23917) and 0.04% (w/v) Pluronic^TM^ F-127 (ThermoFisher, P6867) for 45 min at 37 °C. Excess dye was removed by washing with HBSS. Imaging was carried out using confocal microscope (OLYMPUS IX83-FV3000-OSR) with an excitation wavelength of 494 nm and an emission wavelength of 516 nm by OLYMPUS FV31S-SW software. Odorant solution (11-odorant mixture: isomenthone, citronellol, isoamyl acetate, methyl salicylate, acetophenone, L-carvone, R-carvone, citral, ethyl butyrate, valeraldehyde and β-damascone, each at 100 μM) was applied by bath application. Acquired sequences of images were converted to ΔF/F0, where ΔF was the real-time fluorescent intensity change relative to F0, and F0 was the averaged baseline fluorescence values before odorant onset. Colonies of interest were labeled with a fixed area of region of interest (ROI) using Image J software and fluorescence change over time in this ROI was calculated using GraphPad Prism software.

### Quantification and statistical analysis

Cell counting from the confocal images was performed using Image J software. Ratio of positively stained cells in each colony was defined as the number of positively stained cells versus the number of DAPI^+^ cells. Counting was carried out by someone who was blinded to the experimental conditions design to eliminate bias. Data were presented as mean ± SEM from at least three independent experiments. Band intensity in western blot analysis was measured by Image J software. Statistical difference between two groups was measured by unpaired t test, and among multiple groups was determined by one-way ANOVA or two-way ANOVA using GraphPad Prism 6.0 software.

## Results

### Lgr5^+^ cells sorted from the OE form functional colonies *in vitro*

**Figure 1:**
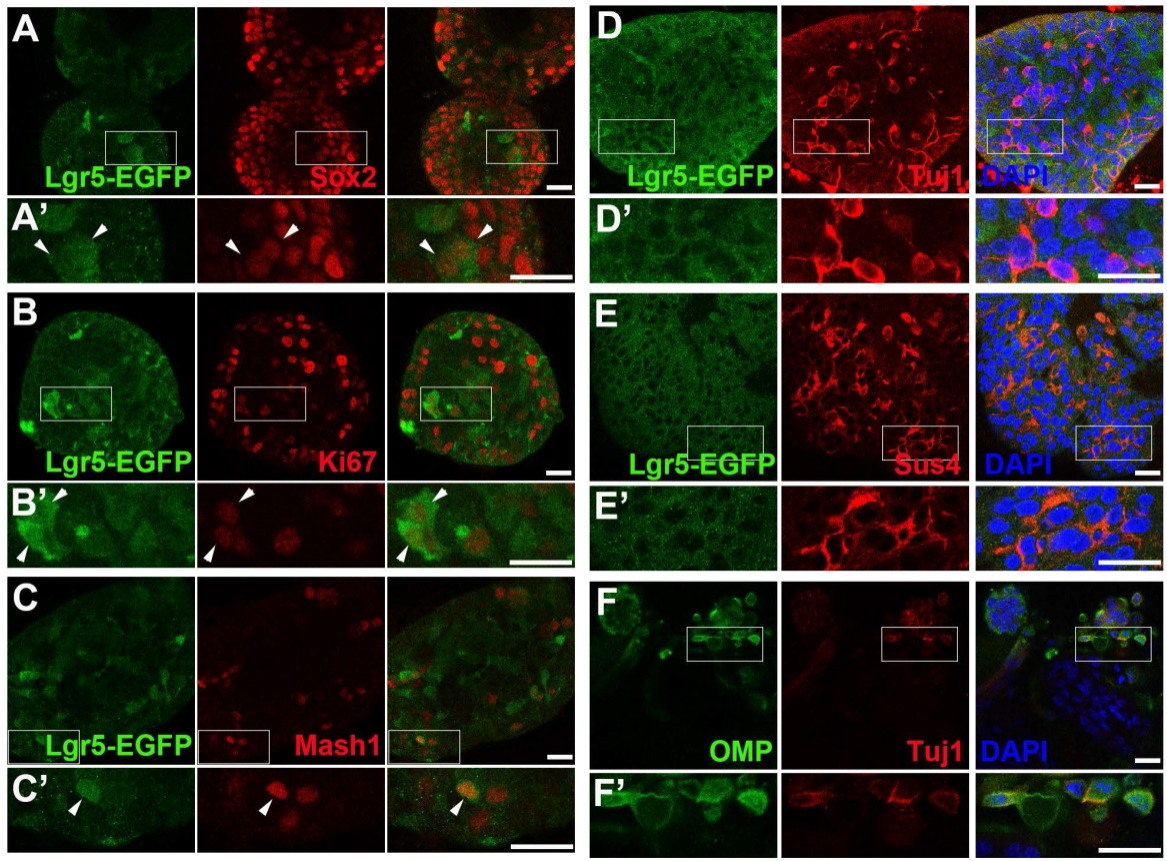
Lgr5-EGFP^+^ cells sorted from the OE of Lgr5-EGFP-CreERT2 mice formed colonies *in vitro*. Confocal images of immunostaining against GFP and Sox2 (A), Ki67 (B) and Mash1 (C) on Day 10 after *in vitro* culture. The squared regions in A-C were shown as A'-C'. The double positively stained cells were marked by arrowheads in A'-C'. (D, E) Immunostaining against GFP and Tuj1, GFP and Sus4 in OE colonies cultured in differentiation medium on Day 10. (F) Immunostaining against Tuj1 and OMP in colonies on Day 21 post differentiation. The squared regions in D-F were shown as D'-F'. Scale bars were 20 µm.

**Figure 2:**
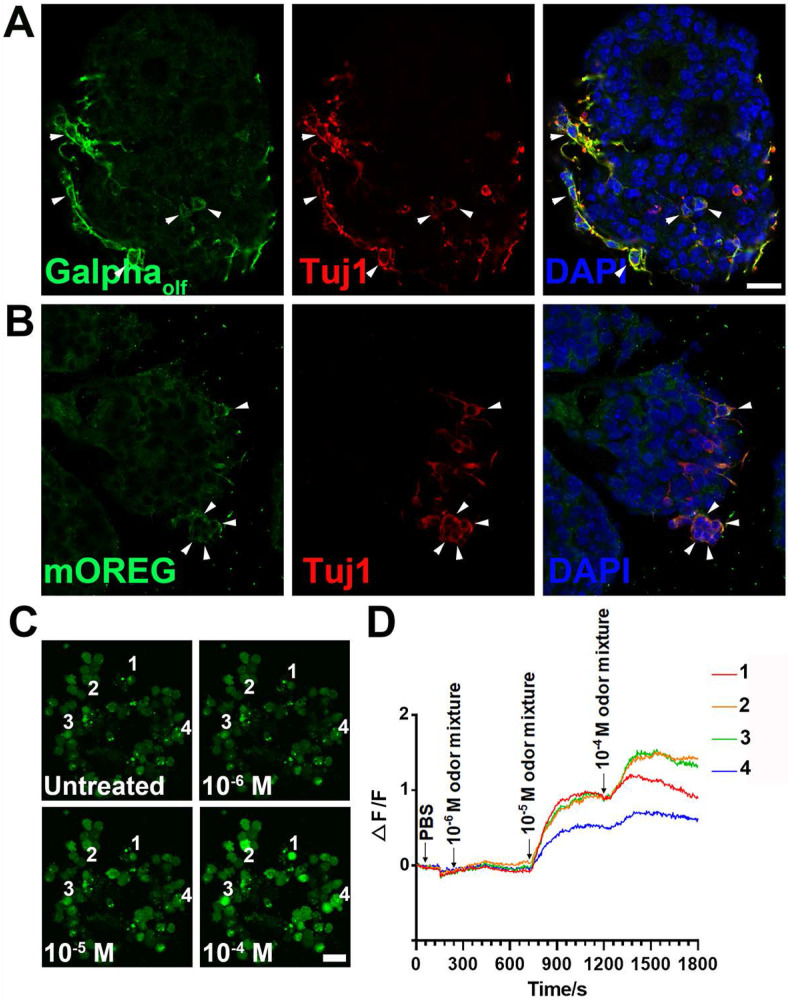
Functional analysis of OE colonies. (A) Confocal images of OE colonies immunostained with antibodies against Galpha_olf_ and Tuj1. (B) Co-immunostaining analysis against olfactory receptor mOREG and neuronal marker Tuj1. Arrowheads indicated double positively stained cells. (C) Confocal images of Fluo-4 AM-loaded OE colonies before (untreated) and after odor mixture stimulation at 10^-6^, 10^-5^ and 10^-4^ M. (D) OE colonies showed elevated Ca^2+^ signals (measured as ΔF/F) in response to 10^-5^ and 10^-4^ M odor mixture, but did not respond to saline or 10^-6^ M odor mixture. Scale bars: 20 µm.

To establish the OE colony culture *in vitro*, we cultured Lgr5-EGFP^+^ cells sorted from the OEs of Lgr5-EGFP-CreERT2 mice. On Day 10 after *in vitro* culture, typical sphere-like structures were observed, similar to cultures obtained from liver and taste Lgr5^+^ progenitor cells 29, 37. Lgr5-EGFP^+^ cells in colonies expressed progenitor cell marker Sox2 (Figure 1A, arrowheads in Figure 1A'), proliferative cell marker Ki67 (Figure 1B, arrowheads in Figure 1B') and neuron-specified basal cell marker Mash1 (Figure 1C, arrowhead in Figure 1C'). Statistically, 47.9 ± 4.2%, 35.2 ± 11.5% and 30.3 ± 8.1% of Lgr5-EGFP^+^ cells in OE colonies were Sox2^+^, Ki67^+^ and Mash1^+^, respectively. When cultured in differentiation medium, Lgr5 expression in OE colonies was diminished on Day 10 after *in vitro* differentiation (Figure 1D). Meanwhile, we observed the presence of Tuj1^+^ (Figure 1D, 1D') and Sus4^+^ (Figure 1E, 1E') cells, indicating the generation of neuronal and supporting cell lineages in cultured colonies. In each colony, 8.6 ± 1.4% of cells were Tuj1^+^ while Sus4^+^ cells accounted for 8.4 ± 2.1%. Furthermore, on Day 21 after differentiation, we found that OMP^+^ cells were scattered (Figure 1F, 1F'), demonstrating that mature olfactory sensory neurons (OSNs) were generated in OE colonies derived from Lgr5^+^ cells. OMP^+^ cells accounted for 14.9 ± 2.4% per colony and 5.3 ± 1.2% of cells were OMP^+^/Tuj1^+^. Furthermore, 70 ± 5% of Tuj1^+^ neurons in OE colonies expressed Galpha _olf_, the alpha unit of specific G protein in OSN (Figure 2A, labeled by arrowheads). We also found that Tuj1^+^ neurons in OE colonies expressed mOR-EG, also known as Olfr73 and MOR174-9, a mouse OR detecting eugenol and a wide range of chemical structures 38, 39 (Figure 2B, labeled by arrowheads). Furthermore, Fluo-4 AM-loaded cells in OE colonies responded to 10^-5^ and 10^-4^ M odorant mixture, but not to 10^-6^ M mixture or saline (Figure 2C-D), indicating that these colonies were functional for odor recognition. Thus, these data indicated that *in vitro*-cultured colonies from Lgr5^+^ cells in the OE had capacity to produce the sustentacular and functional neuronal lineages.

### CHIR99021 promotes Lgr5 expression in OE colonies

**Figure 3:**
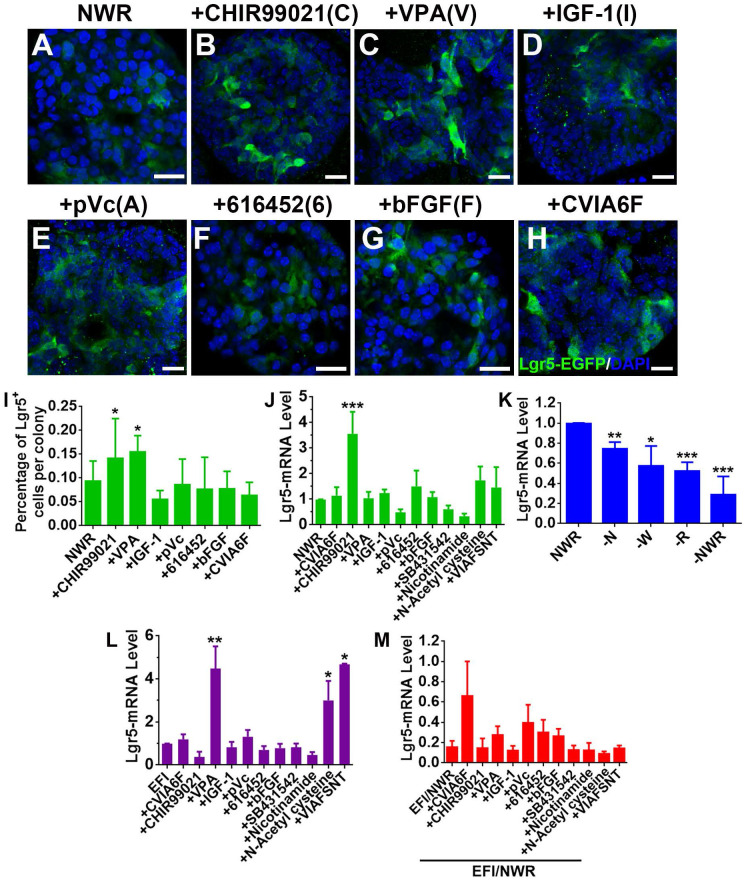
Chemicals altered Lgr5 expression in OE colonies. (A-H) Confocal images of Lgr5-EGFP^+^ cells in OE colonies cultured in NWR-based medium (A), supplemented with CHIR99021 (B), VPA (C), IGF-1 (D), pVc (E), 616452 (F), bFGF (G) and CVIA6F cocktail (H). (I) Statistical analysis on the ratio of Lgr5-EGFP^+^ cells per colony in NWR-based medium with different chemical treatments. (J) Quantitative PCR showed differential Lgr5-mRNA levels in OE colonies under stimulations of various single chemicals and cocktails CVIA6F and VIAFSNT. (K) Quantitative PCR analysis on Lgr5-mRNA level in OE colonies cultured in the absence of Wnt3a, Noggin, R-Spondin 1 or of NWR. (L) The Lgr5-mRNA level in OE colonies cultured in EFI-based medium supplemented with single chemicals and cocktails CVIA6F and VIAFSNT. (M) The ratio of Lgr5-mRNA level between colonies cultured in EFI- and NWR-based media, supplemented with single chemicals and cocktails CVIA6F and VIAFSNT. The statistical difference was determined by unpaired t test. ns, not significant, *p < 0.05, **p < 0.01, ***p < 0.001. Scale bars were 20 µm. Abbreviations of chemicals were as follows: CHIR99021—C, VPA -V, IGF-1—I, pVc—A, 616452—6, bFGF—F, SB431542—S, Nicotinamide—N, N-Acetyl cysteine—T.

Drug cocktail could enhance clonal expansion of Lgr5^+^ cells from mammalian cochlea 30. We hereby tested the effect of a set of chemicals on Lgr5 expression in OE colonies. Single stimulation by CHIR99021 (C) (an inhibitor of GSK-3β and a Wnt activator 40) and VPA (V) (a histone deacetylase (HDAC) inhibitor 41, 42) increased the ratio of Lgr5-EGFP^+^ cells at Passage 2 compared to NWR control (Figure 3A-C, 3I, Figure S1A, S1D, *p <* 0.05). In colonies at Passage 3 and Passage 4, CHIR99021 treatment also led to increases in the ratio of Lgr5-EGFP^+^ cells (Figure S1B-D, *p <* 0.05 at P3 and *p <* 0.001 at P4), while addition of VPA did not significantly increase the ratio of Lgr5-EGFP^+^ cells at Passage 3 (Figure S1B-D, *p >* 0.1 at P3 and *p <* 0.05 at P4). This was also confirmed by the quantitative PCR, showing that CHIR99021 treatment enhanced Lgr5-mRNA level by 2.6 ± 0.8 fold compared to NWR control (Figure 3J, *p <* 0.001, n = 4). Besides, colonies were treated with other chemicals including IGF-1 (I) and bFGF (F) (two basic factors maintaining the growth of Lgr5^+^ cells from mammalian cochlea 30), 616452 (6) (transforming growth factor-β (TGF-β) type I receptor kinase inhibitor 43), pVc (A) (a stable form of vitamin C and promoting cochlear Lgr5^+^ cell expansion 30), SB431542 (S) (an inhibitor of the TGF-β/Activin/NODAL pathway 44), Nicotinamide (N) (the amide form of vitamin B3 and used as a medium supplement in organoid culture 45, 46) and N-Acetyl-L-cysteine (T) (a component of the expansion medium for mouse intestinal stem cell culture 47). However, these chemicals did not significantly change Lgr5-mRNA level in OE colonies cultured in NWR-based medium (Figure 3J). Chemical cocktail CVIA6F (CHIR99021+VPA+IGF-1+pVc+616452+bFGF) and VIAFSNT (VPA+IGF-1+pVc+bFGF+SB431542 +Nicotinamide+N-Acetyl cysteine) also did not drastically increase Lgr5-mRNA level (Figure 3J). Thus, Lgr5 expression in NWR medium-cultured OE colonies was promoted with treatment of CHIR99021, potentially targeting on GSK-3β inhibition.

### Noggin/Wnt3a/R-Spondin 1(NWR) are necessary to maintain Lgr5 expression

The culture medium maintaining OE colony growth contained Noggin (a bone morphogenetic proteins inhibitor 48), Wnt3a (a member of Wnt protein family regulating cell fate 49) and R-Spondin 1 (a specific ligand to Lgr5 50 to promote canonical Wnt/β-catenin signaling 51). To show the necessity of NWR in maintaining Lgr5 expression, one of the three factors was removed from NWR-based culture medium. The Lgr5-mRNA level in OE colonies was decreased by 25 ± 3% (*p <* 0.01, n = 5) without Noggin, by 43 ± 9% (*p <* 0.05, n = 5) without Wnt3a, and by 48 ± 4% (*p <* 0.001, n = 5) without R-Spondin 1 (Figure 3K). Besides, the absence of Noggin, Wnt3a and R-Spondin 1 supplements reduced Lgr5-mRNA level by 71 ± 8% (-NWR, Figure 3K, *p <* 0.001). Thus, NWR were required to maintain Lgr5 expression in OE colonies. To further demonstrate the necessity of NWR, we measured Lgr5-mRNA level in the presence of EFI instead of NWR. EGF, bFGF and IGF-1 are three basic factors maintaining the growth of Lgr5^+^ cells from mammalian cochlea *in vitro* 30. Compared to NWR, presence of EFI significantly decreased Lgr5-mRNA level to 16 ± 6% (Figure 3M, shown as EFI/NWR). In EFI medium-cultured colonies, VPA treatment enhanced Lgr5-mRNA level by 4.3 ± 1.5 fold (Figure 3L, *p <* 0.01, n = 4). Besides, N-Acetyl cysteine and VIAFSNT cocktail increased Lgr5-mRNA level by 2.5 ± 0.9 and 3.6 ± 0.1 fold, respectively (Figure 3L, *p <* 0.05, n = 3). However, compared to NWR-based medium with chemical supplements, culturing in EFI-based medium with corresponding single chemicals and VIAFSNT cocktail showed significant reduction in Lgr5-mRNA levels (Figure 3M, *p <* 0.001), suggesting that EFI-based medium was not optimal for maintaining Lgr5 expression in OE colonies. Thus, we concluded that supplementing NWR into culture medium was preferable to maintain Lgr5 expression in OE colonies.

### CHIR99021 promotes colony growth while VPA enhances progeny generation from Lgr5^+^ cells in OE colonies

Since single chemical could regulate Lgr5 expression in OE colonies, we determined if colony growth was affected by chemical treatment. Treatment with CHIR99021 and 616452 significantly increased colony number per well by 90 ± 22% (*p <* 0.05) and 193 ± 32% (*p <* 0.01), respectively, while other chemicals did not change colony number (Figure S2A). Besides, CHIR99021 increased colony size by 40 ± 7% (Figure S2B, *p <* 0.001). Immunostaining against Ki67 demonstrated that addition of CHIR99021 and 616452 significantly increased the ratio of Ki67^+^ cells by 175 ± 37% (*p <* 0.001) and 82 ± 22% (*p <* 0.01) compared to NWR control (Figure S2C-E, S2I), while CVIA6F cocktail reduced the ratio of Ki67^+^ cells (Figure S2C, S2K). Thus, CHIR99021 promoted growth and proliferation in OE colonies.

Lgr5 marked progenitors/stem cells in cultured OE colonies (Figure 1A-C). We then determined whether progeny generation from Lgr5^+^ cells were affected by chemical treatments mentioned above. Through lineage tracing in colonies derived from the OE of Lgr5-CreERT2/Rosa26-TdTomato mice by 4-Hydroxytamoxifen induction (Figure S3A), we found that stimulation with 616452 decreased the ratio of TdT^+^ cells-containing colonies by 20 ± 5% (Figure S3D, S3H, *p <* 0.05) while VPA led to an increase by 22 ± 2% compared to NWR control (Figure S3B, S3E, S3H, *p <* 0.05), demonstrating that these two chemicals affected generation of progenies from Lgr5^+^ cells. However, treatment with CHIR99021, pVc or bFGF did not significantly alter the ratio of TdT^+^ cells-containing colonies (Figure S3C, S3F, S3G, S3H). Thus, VPA enhanced progeny generation from Lgr5^+^ cells in OE colonies.

### LY411575 enhances sensory neuronal generation in OE colonies

**Figure 4:**
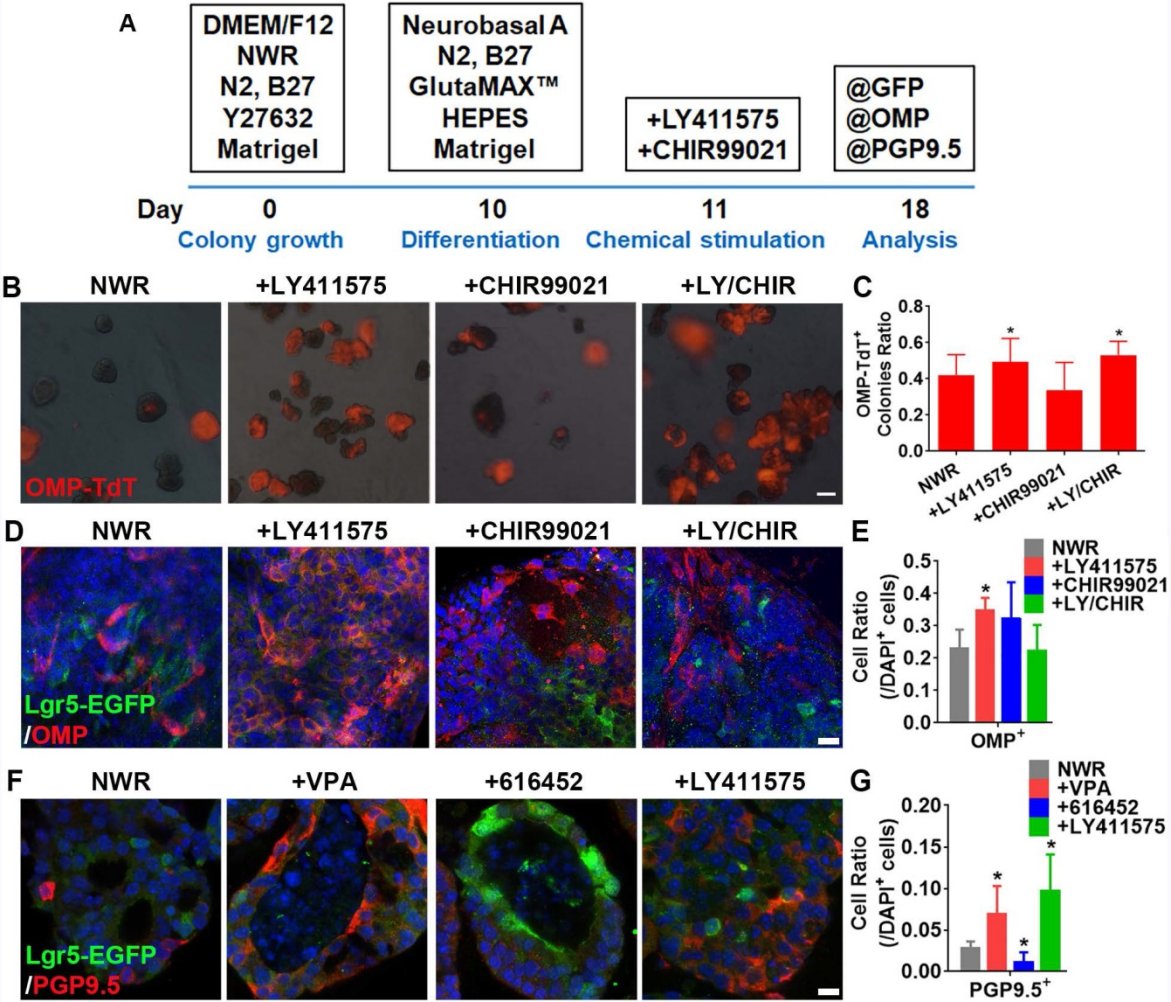
LY411575 promoted generation of OMP^+^ mature sensory neurons. (A) Scheme showing the protocol of colony differentiation. (B) Images of colonies derived from OMP-Cre/Rosa26-TdTomato mice with treatments of CHIR99021, LY411575 and CHIR99021/LY411575. (C) Statistical analysis on the ratio of OMP-TdT^+^ colonies with treatments of CHIR99021, LY411575 and CHIR99021/LY411575. (D) Confocal images of OMP^+^ cells in OE colonies stimulated with CHIR99021, LY411575 and CHIR99021/LY411575. (E) Statistical analysis on the ratio of OMP^+^ cells in OE colonies treated with CHIR99021, LY411575 and CHIR99021/LY411575. (F) Confocal images of PGP9.5^+^ cells in OE colonies treated with VPA, 616452 and LY411575. (G) Statistical analysis on the ratio of PGP9.5^+^ cells under stimulations of VPA, 616452 and LY411575. The statistical difference was determined by one-way ANOVA with Dunnett's multiple comparisons test. *p < 0.05. Scale bar(s) in (B) was 200 µm, in (D) and (F) were 10 µm.

Previous report indicated LY411575 (a γ-secretase inhibitor and Notch inhibitor to promote the neuronal differentiation 52, 53) plus CHIR99021 led to robust generation of hair cells in Lgr5^+^ colonies from mammalian cochlea 30. Here, we determined whether these chemicals affected sensory neuronal generation in OE colonies. Protocol of sensory neuronal differentiation was shown in Figure 4A. Apparent change in the ratio of OMP-TdT^+^ colonies cultured in NWR-based medium was observed with LY411575 and LY411575/CHIR99021 treatments, leading to increases by 18 ± 7% and 26 ± 5%, respectively (*p <* 0.05, Figure 4B-C). Through immunostaining against mature OSN marker OMP, we found a robust increase in the ratio of OMP^+^ cells by 57 ± 10% in LY411575-treated colonies compared to the ratio in untreated colonies (*p <* 0.05, Figure 4D-E). Besides, LY411575 treatment also increased the percentage of PGP9.5^+^ cells by 2.5 ± 0.6 fold (*p <* 0.05, Figure 4F-G), while 616452 drastically decreased the ratio of PGP9.5^+^ cells by 61 ± 19% compared to NWR control (*p <* 0.05, Figure 4F-G). Thus, Notch inhibitor LY411575 promoted sensory neuronal generation in colonies derived from Lgr5^+^ cells in the OE.

### EFI induces morphological alteration in OE colonies

Since culturing in EFI-based medium altered Lgr5 expression level, we explored if OE colonies in EFI-based medium underwent morphological change from filled to cystic appearance, which was associated with differentiation in intestine colonies 54. Single chemicals and drug cocktails were added to evaluate their effects on colony morphology. In the presence of EFI-based medium, single chemicals such as pVc, SB431542 and N-Acetyl cysteine drastically increased the ratio of colonies with cystic appearance (Figure S4A, S4B, *p <* 0.001). Notably, chemical cocktail -VPA (IAFSNT, removal of VPA from VIAFSNT cocktail) changed the morphology most significantly, while other cocktails such as -IGF-1, -bFGF and -SB431542 also increased the ratio of cystic colonies compared to EFI control (Figure S4A, S4C, *p <* 0.001). In the absence of pVc (-pVc), the ratio of cystic colonies was significantly decreased compared to that in VIAFSNT-treated colonies (Figure S4A, S4C, *p <* 0.05, noted by green asterisk). When colonies were cultured in NWR-based medium, only N-Acetyl cysteine (Cys) enhanced the ratio of cystic colonies, while other single chemicals did not significantly induce morphological alteration (Figure S4E, S4F, *p <* 0.001). By comparison, chemical cocktails such as VIAFSNT (*p <* 0.001), -VPA (*p <* 0.05), -IGF-1 (*p <* 0.01), -bFGF (*p <* 0.001) and -Nicotinamide (*p <* 0.001) significantly increased the ratio of cystic colonies in contrast to NWR control (Figure S4E, S4G). However, compared to the VIAFSNT, removal of one chemical from cocktail drastically decreased the ratio of cystic cultures except for removal of bFGF (Figure S4E, S4G, *p <* 0.01 for -Nicotinamide, *p <* 0.001 for other cocktail treatments, noted by green asterisks). Culturing in EFI-based medium increased the colony size by 47 ± 6% compared to in NWR-based medium. Chemical treatments such as Cys, IAFSNT (-VPA) and VIAFSNT did not significantly alter the colony size in contrast to NWR control (Figure S4H), while Cys and IAFSNT (-VPA) treatments with EFI-based medium increased the colony size by 1.2 ± 0.2 and 2.3 ± 0.1 fold compared to EFI control (*p <* 0.001, Figure S4D). Therefore, culturing in EFI-based medium significantly induced the generation of OE colonies with cystic appearance.

**Figure 5:**
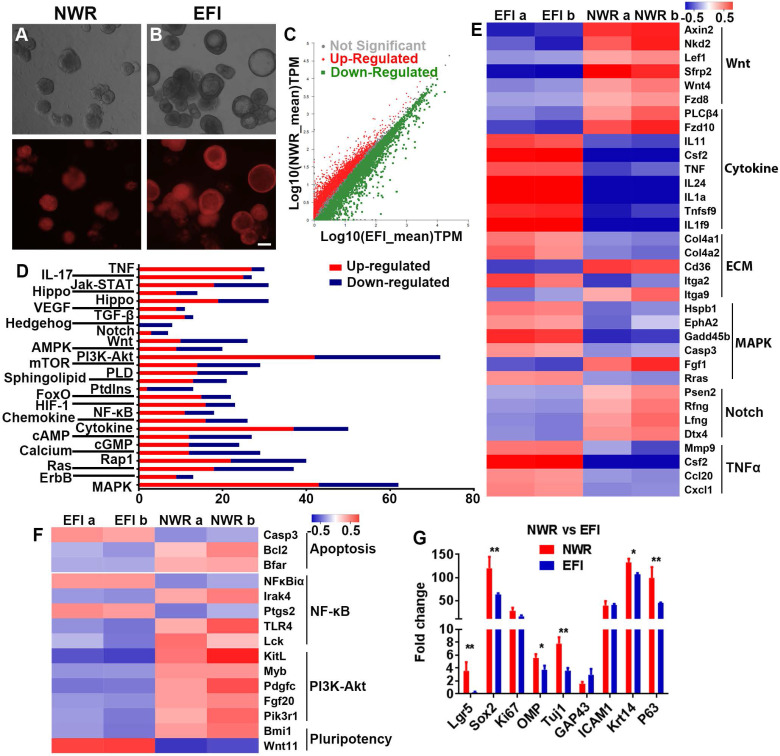
Transcriptional analysis of colonies (with both filled and cystic appearance) cultured in NWR- and EFI-based media. (A, B) Images of OMP-TdT^+^ colonies in NWR- and EFI-based media. (C) Scatter plot of significantly and non-significantly differentially expressed genes between colonies cultured in NWR- and EFI-based media. (D) KEGG enrichment analysis of differentially expressed genes participating in multiple signaling pathways. (E) Heatmap of up- and down-regulated genes in Wnt, Cytokine receptor interaction, ECM, MAPK, Notch and TNFα signaling pathways between colonies cultured in NWR- and EFI-based media. (F) Heatmap of up- and down-regulated genes in apoptosis, NF-κB, PI3K-Akt and pluripotency-associated signaling pathways. (G) Fold change of OE biomarker expression level in colonies cultured in NWR- and EFI-based media. The statistical difference was determined by two-way ANOVA with Sidak's multiple comparisons test. *p < 0.05, **p < 0.01. Scale bar: 100 µm.

We then determined whether the morphological alteration in OE colonies was accompanied by changes at the transcriptional level. Through RNA-seq analysis, colonies cultured between NWR- and EFI-based media (Figure 5A-B) displayed that half of genes (3181 from 6322 genes) were differentially expressed (Figure 5C). KEGG enrichment analysis indicated that these differentially expressed genes were involved in multiple signaling pathways, and the significantly affected signaling pathways include PI3K-Akt, Cytokine receptor interaction, MAPK, etc. (Figure 5D). Heatmap showed the down-regulated genes in Wnt and Notch signaling and up-regulated genes in Cytokine receptor interaction, MAPK and TNFα signaling in EFI medium-cultured colonies compared to genes in NWR medium-cultured colonies (Figure 5E). Meanwhile, genes participating in proliferation-related pathway such as PI3K-Akt were up-regulated in colonies cultured in NWR-based medium (Figure 5F). Multiple OE biomarkers were also up-regulated in colonies cultured in NWR-based medium, such as GBC markers Lgr5 and Sox2, mature and immature sensory neuronal markers OMP and Tuj1, as well as HBC markers Krt14 and P63, when compared to those in colonies cultured in EFI-based medium (Figure 5G). In colonies cultured in NWR-based medium, chemical cocktail VIAFSNT led to significant morphological change (Figure S5A-B) and apparent transcriptional alteration (Figure S5C). These differentially expressed genes participated in multiple signaling pathways including Cytokine receptor interaction, PI3K-Akt, MAPK, etc. (Figure S5D). Most genes in NF-κB, PI3K-Akt, TNFα and MAPK signaling pathways were down-regulated in colonies treated with VIAFSNT compared to genes in untreated colonies (Figure S5E-F). Levels of GBC markers Lgr5 and Sox2 as well as OSN marker OMP were decreased in colonies treated with VIAFSNT (Figure S5G). Furthermore, treatment with IAFSNT (removal VPA from VIAFSNT cocktail) resulted in significant morphological alteration, especially in EFI medium-cultured colonies (Figure S6A, S6D) and experienced drastic transcriptional change (Figure S6B, S6E). These differentially expressed genes participated in multiple signaling pathways (Figure S6C, S6F). The most significantly affected pathway was PI3K-Akt signaling, in which most genes were downregulated in IAFSNT-treated colonies (Figure S6G, S6H). In colonies cultured in NWR-based medium supplemented with IAFSNT, Lgr5 and Tuj1 expression was significantly decreased, while Lgr5, Tuj1, GAP43 and Krt14 were down-regulated in colonies cultured in EFI-based medium with IAFSNT cocktail (Figure S6I, S6J). Collectively, the morphological alteration induced by different base media and drug cocktails was accompanied by transcriptional changes in multiple signaling pathways and alterations in expression levels of OE biomarkers.

### Downregulation of Lgr5 inhibits proliferation in murine OE colonies

**Figure 6:**
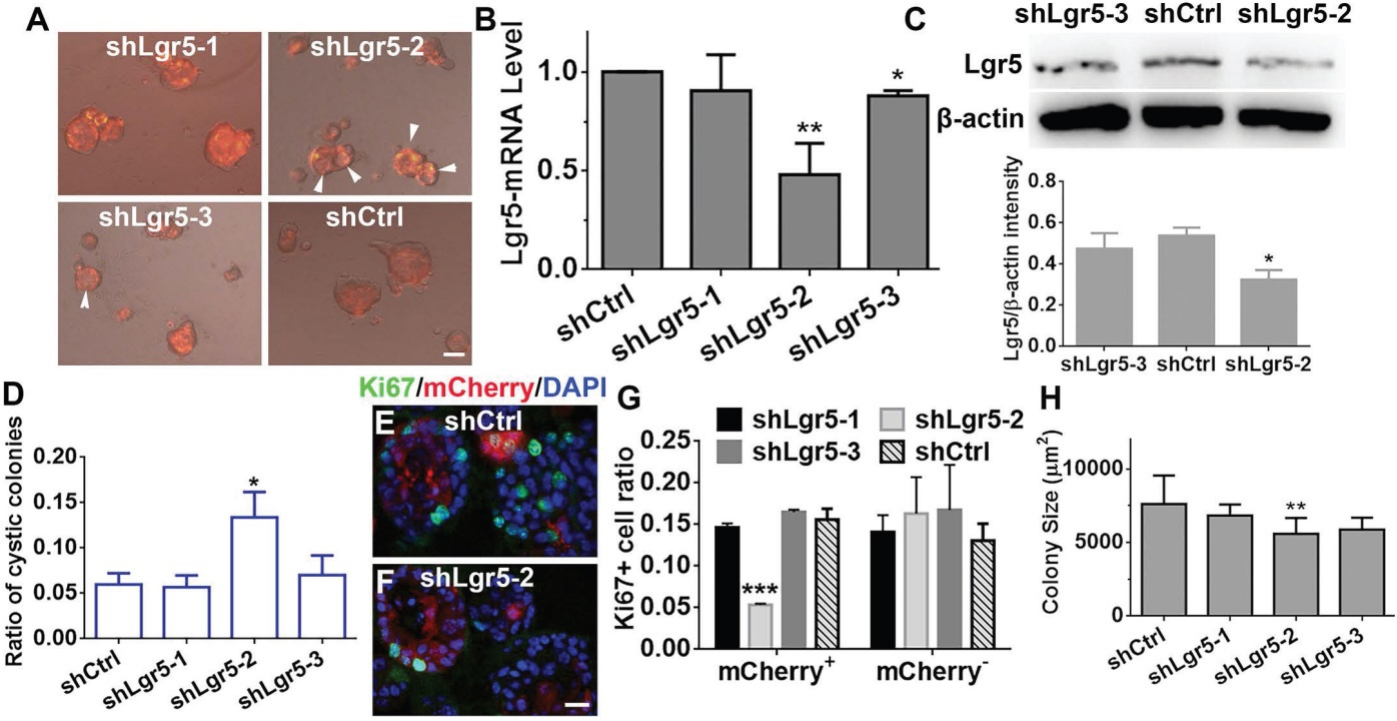
Lgr5 regulated proliferation in OE colonies cultured in NWR-based medium. (A) Images of mCherry^+^ colonies infected with Lenti-shLgr5 and Lenti-shCtrl. Arrowheads labeled cystic colonies. (B) Quantitative PCR analysis of Lgr5-mRNA level in OE colonies infected with Lenti-shLgr5 and Lenti-shCtrl. (C) Western blot showed the significant change of Lgr5 protein level in colonies infected with Lenti-shLgr5-2 compared to the level in colonies infected with Lenti-Ctrl. (D) Analysis on the ratio of cystic colonies infected with Lenti-shCtrl and Lenti-shLgr5. (E, F) Confocal images of Ki67^+^ cells in mCherry^+^ colonies infected with Lenti-shCtrl and Lenti-shLgr5-2. (G) Statistical analysis on the ratio of of Ki67^+^ cells in mCherry^+^ and mCherry^-^ colonies infected with Lenti-shLgr5-1, Lenti-shLgr5-2, Lenti-shLgr5-3 and Lenti-shCtrl. (H) Analysis on the size of colonies infected with Lenti-shLgr5 and Lenti-Ctrl. The statistical difference was determined by one-way ANOVA with Dunnett's multiple comparisons test in (B-D, H, L) and by two-way ANOVA with Sidak's multiple comparisons test in (G). *p < 0.05, **p < 0.01, ***p < 0.001. Scale bars, 100 µm in (A), 20 µm in (F).

Considering that Lgr5^+^ cells formed colonies *in vitro*, Lgr5 might play a role in colony proliferation. To demonstrate the role of Lgr5, we infected colonies cultured in NWR-based medium with Lenti-shLgr5-mCherry to downregulate Lgr5 expression. On Day 10 post infection, we observed apparent mCherry^+^ fluorescence in all infected groups (Figure 6A). The efficiency of shLgr5 lentivirus was confirmed by quantitative PCR, indicating Lenti-shLgr5-2 had the most significantly down-regulatory effect on OE colonies (Figure 6B, *p <* 0.01, n = 3). This was further validated by western blot, demonstrating the Lgr5 protein level was significantly reduced in OE colonies infected with Lenti-shLgr5-2 compared to the level in colonies infected with Lenti-Ctrl (Figure 6C, *p <* 0.05, n = 3). Lgr5 downregulation increased the ratio of cystic colonies by 1.8 ± 0.4 fold (Figure 6D, *p <* 0.05, colonies with cystic appearance were noted by arrowheads in Figure 6A). In mCherry^+^ colonies, Lgr5 downregulation lowered the ratio of Ki67^+^ cells by 65 ± 3% (Figure 6E-G, *p <* 0.001). By contrast, there was no significant alteration in the ratio of Ki67^+^ cells between mCherry^-^ Lenti-shCtrl- and Lenti-shLgr5-infected colonies (Figure 6G). The size of colonies infected with Lenti-shLgr5-2 was significantly decreased by 14 ± 3% compared to the size of Lenti-shCtrl-infected colonies (Figure 6H, *p <* 0.01). Thus, these data demonstrated that Lgr5 downregulation hampered proliferation in OE colonies.

### Colony culture from human olfactory mucosa

**Figure 7:**
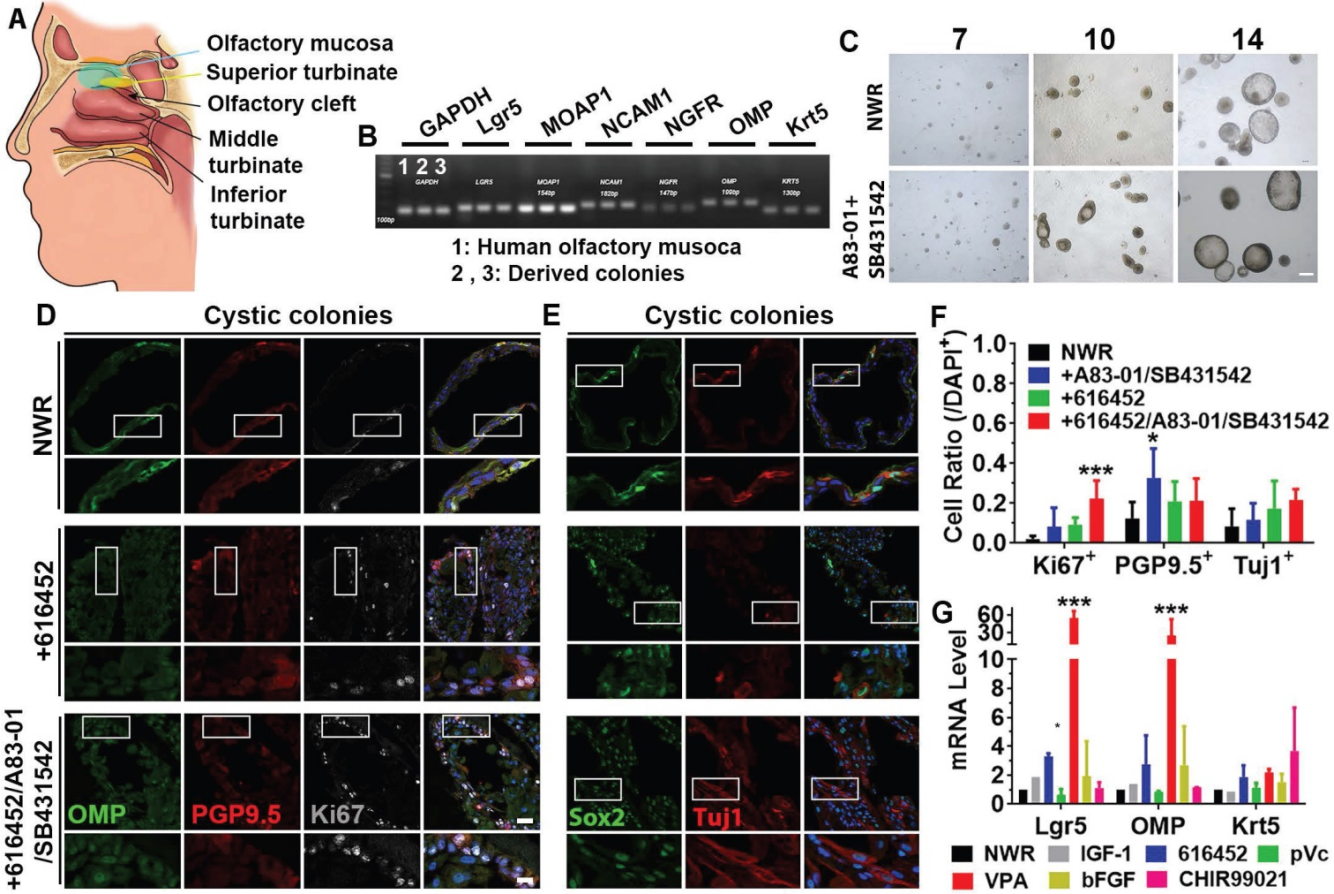
Expansion of colonies from human olfactory mucosa. (A) Diagram showing the human olfactory mucosa, from which colonies were derived. (B) Presence of biomarkers in human olfactory mucosa and derived colonies by RT-PCR. (C) Images of colonies in NWR-based medium with and without treatment of A83-01/SB431542 on Day 7, 10 and 14 post *in vitro* culture. (D, E) Immunostaining against OMP, PGP9.5, Ki67, Sox2 and Tuj1 in human cystic colonies cultured in NWR-based medium, supplemented with 616452 and 616452/A83-01/SB431542. (F) Statistical analysis on the ratios of Ki67^+^, PGP9.5^+^ and Tuj1^+^ cells in human cystic colonies with treatments of 616452, A83-01/SB431542 and 616452/A83-01/SB431542. (G) Quantitative PCR analysis showed Lgr5-, OMP- and Krt5-mRNA levels in human colonies treated with IGF-1, 616452, pVc, VPA, bFGF and CHIR99021. The statistical difference was determined by two-way ANOVA with Sidak's multiple comparisons test. *p < 0.05, ***p < 0.001. Scale bars, 100 µm in (C), 25 µm in (D) and (E), 10 µm in enlarged images from boxed areas.

To further elucidate if the above-mentioned culture condition was applicable on human tissues, we established colony culture from human olfactory mucosae (Figure 7A), isolated from patients undergoing surgery to access tumors on the skull base. Marker genes such as Lgr5, MOAP1, NCAM1, NGFR, OMP and Krt5 were expressed in both olfactory mucosa and derived colonies (Figure 7B). Visible colonies with both filled and cystic appearance were present on Day 7 and typical cystic cultures were observed on Day 14 (Figure 7C). Ttreatment with A83-01 (a TGF-β kinase/activin receptor-like kinase inhibitor to promote cell proliferation in *in vitro* culture expansion 55) and SB431542 (TGF-β RI Kinase Inhibitor VI) increased the colony size by 11 ± 3% on Day 10, but the size was not apparently different on Day 14. Treatment with A83-01 and SB431542 did not significantly change morphology of human colonies (Figure S7A). However, the ratio of cystic colonies was significantly increased on Day 14 compared to Day 10 while the ratio of filled colonies was drastically decreased (Figure S7A, *p <* 0.001). In both cystic and filled colonies, we observed positive staining against neuronal markers PGP9.5, Tuj1 and OMP as well as proliferative progenitor markers Ki67 and Sox2 (Figure 7D, 7E, Figure S7C, S7F).Chemical cocktail of 616452, A83-01 and SB431542 drastically increased the ratio of Ki67^+^ proliferative cells in NWR medium-cultured cystic colonies (Figure 7F, *p <* 0.001), while treatment with A83-01 and SB431542 significantly increased the ratio of PGP9.5^+^ cells in cystic colonies (Figure 7F, *p <* 0.05). However, the ratio of Tuj1^+^ neurons was not significantly changed in cystic colonies under chemical stimulations (Figure 7D-F). Changes in the ratios of Ki67^+^, PGP9.5^+^ and Tuj1^+^ cells in filled colonies were as similar as in cystic colonies with chemical treatments (Figure S7B-H). Meanwhile, we also tested single chemicals from CVIA6F cocktail on human colonies. Addition of VPA (*p <* 0.001) and 616452 (*p <* 0.05) significantly increased Lgr5- and OMP-mRNA levels (Figure 7G). The ratio of Lgr5^+^ cells in human colonies was increased by 66 ± 10% with treatment of 616452 (Figure S8A-B, S8G, *p <* 0.001) and by 44 ± 8% in the presence of VPA (Figure S8C, S8G, *p <* 0.001), but was not significantly changed with treatment of IGF-1, pVc or bFGF (Figure S8D-G). Meanwhile, treatments with 616452 and VPA enhanced the ratio of Ki67^+^ cells by 2.7 ± 0.6 (*p <* 0.01) and 3.7 ± 0.6 fold (*p <* 0.001) (Figure S8A-C, 8G). Therefore, we concluded that colonies derived from human olfactory mucosa was established in defined medium *in vitro*, while the proliferation and differentiation were regulated by inhibition on TGF-β receptor kinase and HDAC.

## Discussion

In this study, we explored chemicals facilitating the expansion of Lgr5^+^ colonies from the murine OE and found that CHIR99021 and VPA induced highest Lgr5-mRNA level in colonies cultured in NWR- and EFI-based media, respectively. LY411575 significantly enhanced sensory neuronal generation in murine OE colonies. Culturing with EFI-based medium led to cystic appearance of murine colonies and was accompanied by significant alteration in transcriptomes involved in multiple signaling pathways. In colonies from human olfactory mucosa, addition of VPA and 616452 was favorable to Lgr5 expression and cell proliferation, while A83-01/SB431542 promoted neuronal differentiation. Thus, this study optimized the culture condition of olfactory epithelium/mucosa colony culture, potentially providing a new source for cell-based transplantation therapy against smell loss.

Lgr5^+^ cells from multiple tissues form 3D cultures *in vitro* with regulation of Wnt signaling and through HDAC inhibition. CHIR99021 and VPA enhanced self-renewal of Lgr5^+^ stem cells in mouse small intestinal colonies 33 and led to significant expansion of cochlear Lgr5^+^ supporting cells 30. Consistent with these findings, VPA and CHIR99021 significantly enhanced the ratio of Lgr5-EGFP^+^ cells in colonies derived from Lgr5^+^ cells in the murine OE while Lgr5-mRNA level was increased by CHIR99021 in NWR-based medium (Figure 3I-J). Besides, VPA treatment increased Lgr5-mRNA level in OE colonies cultured in EFI-based medium (Figure 3L), potentially suggesting that HDAC inhibition may synergize with EFI to activate Lgr5 expression. Same chemical differentially regulated Lgr5 expression in murine OE colonies cultured in EFI- and NWR-based media, typically exampled by CHIR99021 and VPA (Figure 3J-L). CHIR99021 acts as an inhibitor of GSK-3β and functions as a Wnt activator 40. Thus, it may potentiate the effect of Wnt3a to activate Wnt signaling. Meanwhile, we added R-Spondin 1, a specific ligand to Lgr5 50 to promote canonical Wnt/β-catenin signaling 51, into colony growth medium. Therefore, it is logical that addition of CHIR99021 into NWR-based medium increases the Lgr5-mRNA level in OE colonies. VPA is a HDAC inhibitor, potentially functioning as a Notch signaling activator 41, 42. EGF and Notch signaling exhibited synergic effect to exert multiple biological functions 56-58. In addition, bFGF was reported to activate stem cell proliferation 59 and inhibit progenitor differentiation via Notch signaling pathway 60. These finding demonstrated the crosstalk between EGF/bFGF and Notch signaling, potentially explaining the increase in Lgr5-mRNA level in OE colonies cultured in EFI-based medium with addition of VPA. However, the specific mechanisms underlying the effects of NWR + CHIR99021 and EFI + VPA on Lgr5-mRNA level still need further exploration.

Cystic structure is a typical morphology in organoids 61, especially for organoids from human tissues 62, 63. The significance of morphological alteration has not been clearly determined yet. In the current work, we found that most murine OE colonies cultured in NWR-based medium had the 'filled' morphology, while apparent cystic morphology appeared in the presence of EFI-based medium (Figure S4). More significantly, colonies in EFI-based medium showed reduction in Lgr5-mRNA level compared to colonies in NWR-based medium (Figure 3M), suggesting morphological transition was likely to be associated with the alteration in critical signaling pathways. This was confirmed by RNA-seq analysis, showing that different base media and chemical cocktails induced significant change in transcriptomes participating in multiple signaling pathways including PI3K-Akt, Cytokine receptor interaction, MAPK, etc. (Figure 5, S5, S6). Previous reports showed that human pluripotent stem cell-derived intestinal organoids underwent maturation with morphological alteration 64. Morphological variation in Lgr5^+^ embryonic liver cells-formed organoids represented different cellular subtypes 65. Combining our RNA-Seq and Lgr5 downregulation experiments, cystic OE colonies in EFI-based medium showed reduction in Lgr5-mRNA level while Lgr5 downregulation in OE colonies led to significant increase in the ratio of cystic colonies and reduction in the ratio of Ki67^+^ cells (Figures 5-6). These data demonstrated that Lgr5 expression was associated with the morphological alteration, potentially mediating proliferation in OE colonies.

Human olfactory stem cells under 2D culture are promising in the treatment of degenerative disease 26, 27 and for nerve reconstruction 25, 66. This indicates the potential clinical significance of human olfactory stem cells. However, it is still unclear whether 2D culture system is suitable for self-renewal of Lgr5^+^ stem cells from other organs 67. With the rapid advancement of 3D culture system, organoid culture technology has raised enormous expectations through growing human tissues in a dish 68. Using Matrigel-based 3D culture, we hereby established colonies from human olfactory mucosa under chemically defined conditions (Figure 7, S7, S8). Chemical cocktail used in human colonies culture was different from those in mouse colonies. 616452 is a transforming growth factor-β (TGF-β) type I receptor kinase inhibitor 43. A83-01 is a TGF-β kinase/activin receptor-like kinase (ALK 5) inhibitor that prevents phosphorylation of Smad2/3 69. SB431542 is an inhibitor of the TGF-β/Activin/NODAL pathway that inhibits ALK5, ALK4 and ALK7 through inhibiting SMAD2 phosphorylation 44. These suggest that TGF-β signaling is important in maintaining growth of colonies from the human olfactory mucosa. Considering that organoids from patients can be used for high-throughput drug screening 70 and disease modeling 71, our study provided ideal source for cell transplantation therapy as well as for drug and genetic screens for nasal diseases. Furthermore, this work suggested that chemical cocktail could be a potential therapeutic route to restore the olfactory sensory neurons through regulating differentiation of olfactory progenitor cells. However, more efforts will be made on the functional assays to verify these colonies from murine OE and human olfactory mucosa were real 'organoids'.

In summary, this work established culture of murine and human colonies from nasal epithelium and olfactory mucosa under defined chemical treatments, potentially providing source to stem cell therapy against loss of smell caused by sensory neuronal degeneration.

## Supplementary Material

Supplementary figures.

## Abbreviations

OE: olfactory epithelium
HBC: horizontal basal cells
GBC: globose basal cells
Lgr5: Leucine-rich repeat-containing G-protein coupled receptor 5
bFGF: basic fibroblast growth factor
EGF: epidermal growth factor
IGF-1: insulin-like growth factor 1
VPA: Valproic acid
pVc: L-ascorbic acid 2-phosphate
NWR: Noggin/Wnt3a/R-Spondin 1
EFI: EGF/bFGF/IGF-1
OMP: olfactory mature protein
TdT: TdTomato
CVIA6F: CHIR99021/VPA/IGF-1/pVc/616452/bFGF
VIAFSNT: VPA/IGF-1/pVc/bFGF/ SB431542/Nicotinamide/N-Acetyl cysteine
