# Supplementary material for: Expansion of murine and human olfactory epithelium/mucosa colonies and generation of mature olfactory sensory neurons under chemically defined conditions
Source: Theranostics. 2021 Jan 1;11(2):684–99. doi: 10.7150/thno.46750 (PMC7738855; doi:10.7150/thno.46750)
Supplement: Supplementary file 1 — Supplementary figures. [file thnov11p0684s1.pdf]

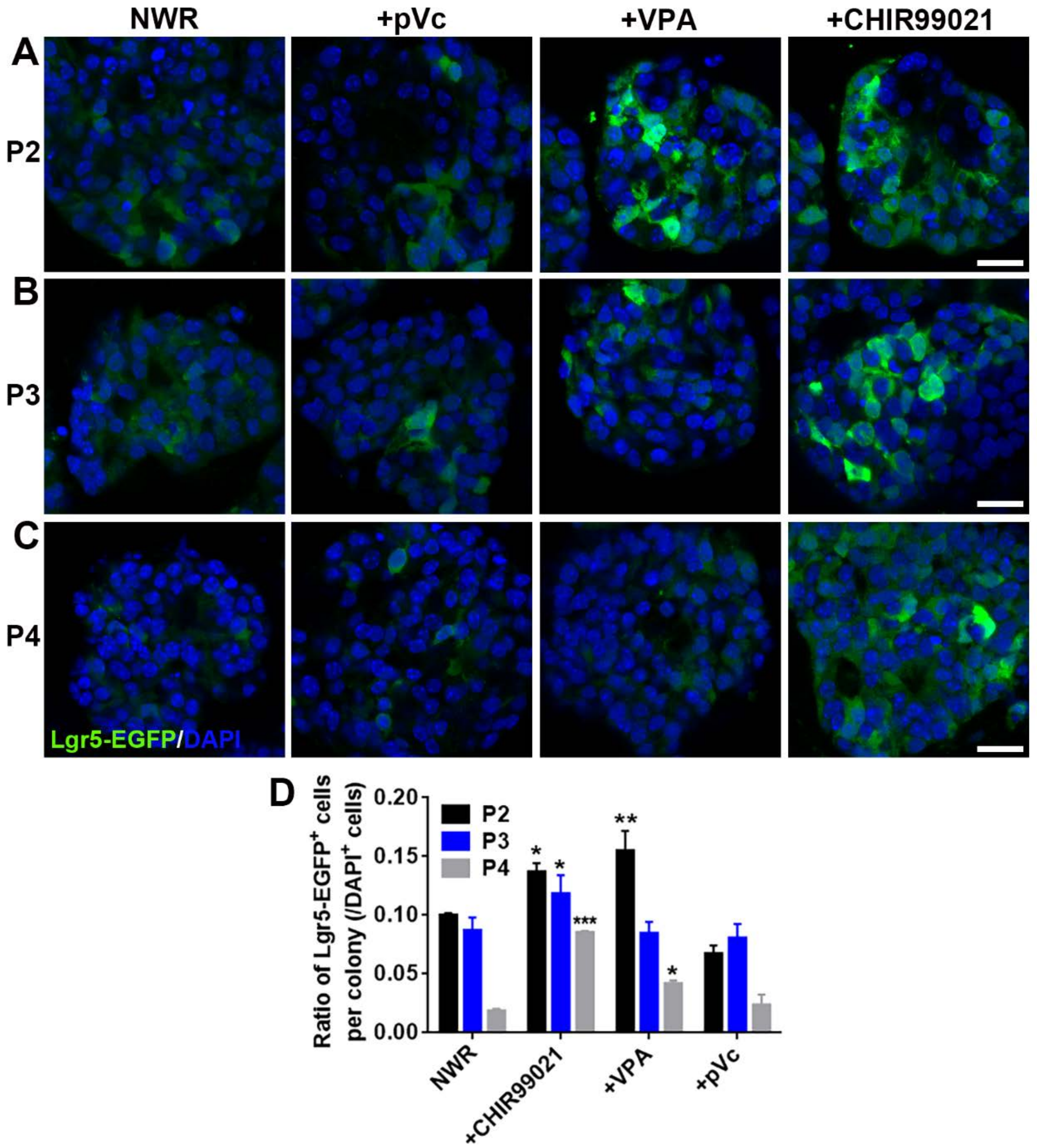

Supplementary Figure 1. Chemicals increased the ratio of Lgr5-EGFP$^+$ cells in OE colonies at various passages. (A-C) Confocal images of Lgr5-EGFP$^+$ cells in murine OE colonies at Passage 2, 3 and 4 under stimulations of pVc, VPA and CHIR99021. (D) Statistical analysis on the ratio of Lgr5-EGFP$^+$ cells in OE colonies at various passages. The statistical difference between untreated (NWR) and chemical-treated colonies at the same passage was determined by unpaired t test. *$p < 0.05$, **$p < 0.01$, ***$p < 0.001$. Scale bars: 20 μm.

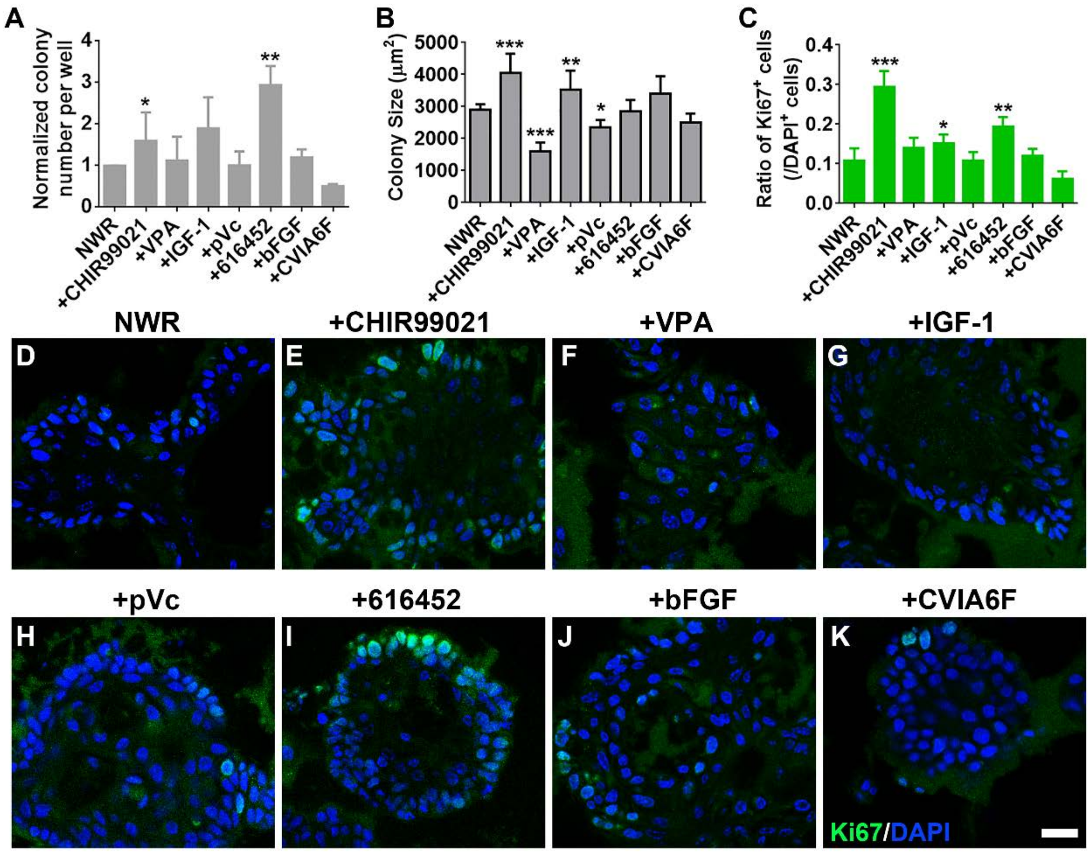

Supplementary Figure 2. Chemical treatments affected colony growth. (A) Statistical analysis on colony number per well under different chemical stimulations. (B) Statistical analysis on the size of colonies treated with different chemicals. (C) Statistical data on the ratio of Ki67$^+$ cells in colonies treated with various chemicals. (D-K) Immunostaining against Ki67 in untreated colonies cultured in NWR-based medium (D) and colonies under stimulations of CHIR99021 (E), VPA (F), IGF-1 (G), pVc (H), 616452 (I), bFGF (J) and CVIA6F cocktail (K). The statistical difference was determined by unpaired t test. *$p < 0.05$, **$p < 0.01$, ***$p < 0.001$. Scale bar was 20 μm.

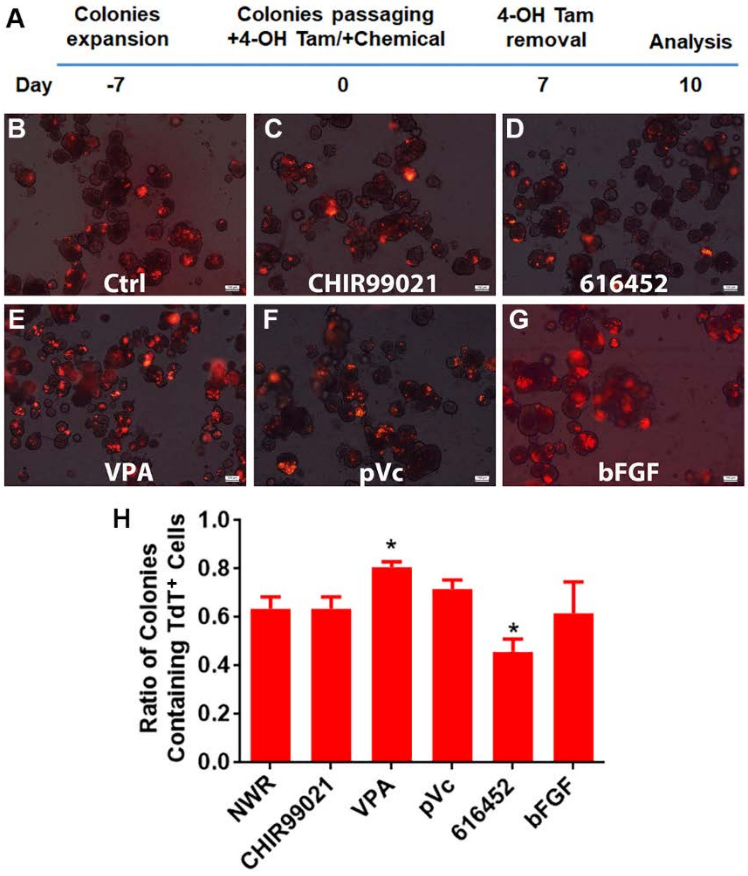

Supplementary Figure 3. Chemical treatments affected progeny generation from $Lgr5^+$ cells in OE colonies. (A) Scheme showing the protocol for lineage tracing of $Lgr5^+$ cells in OE colonies derived from Lgr5-CreERT2/Rosa26-TdTomato mice. (B-G) Images of $TdTomato^+$ (TdT^+) colonies treated with saline (B), CHIR99021 (C), 616452 (D), VPA (E), pVc (F) and bFGF (G). (H) Statistical analysis on the percentage of colonies containing TdT^+ cells. The statistical difference was determined by one-way ANOVA with Dunnett's multiple comparisons test. *$p < 0.05$. Scale bars: 100 μm.

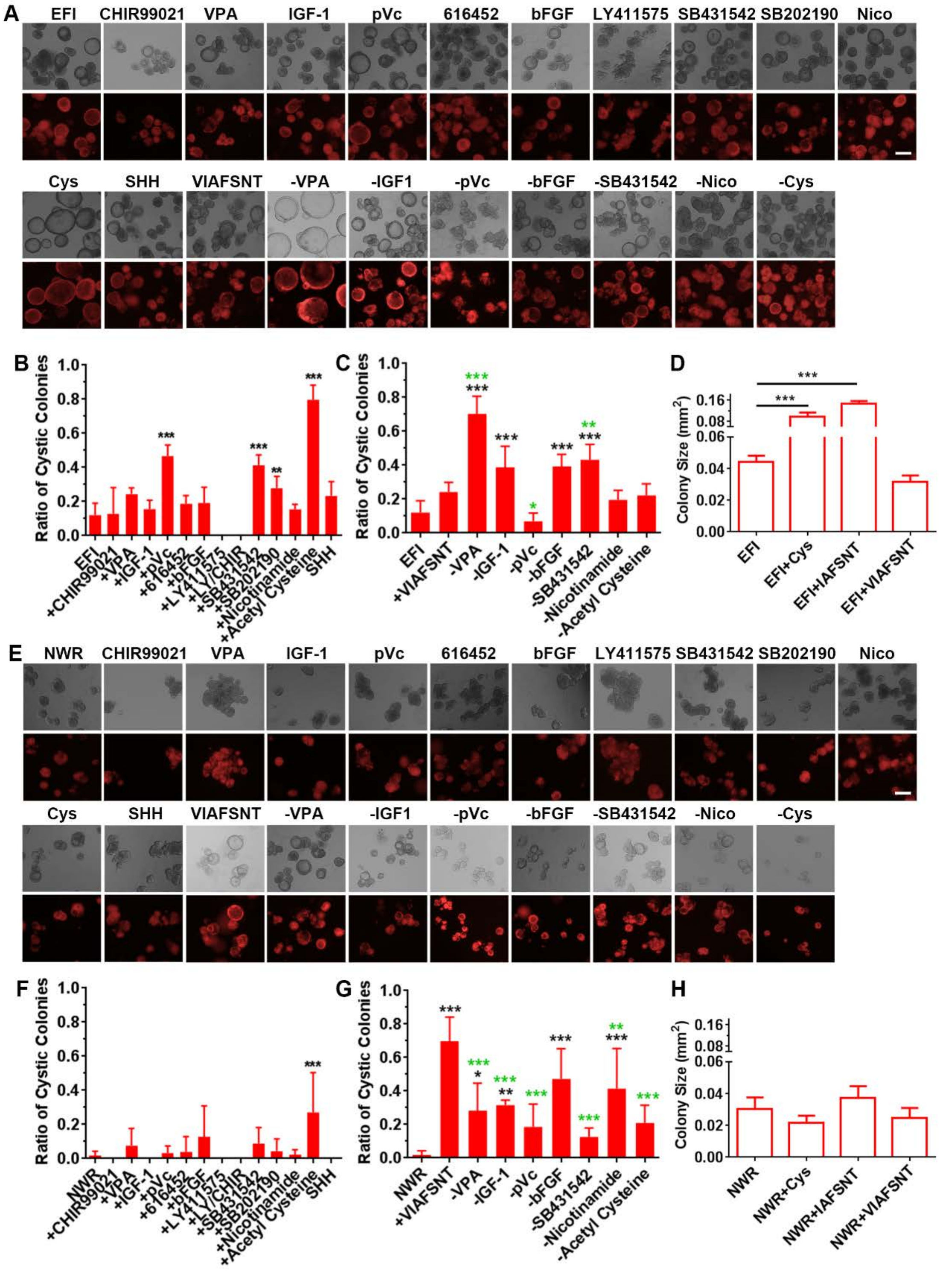

Supplementary Figure 4. Chemical stimulations led to morphological change from filled to cystic appearance in OMP-TdT$^+$ colonies cultured in NWR- and EFI-based medium. (A) Images of OMP-TdT$^+$ colonies in EFI-based culture medium under stimulation of single chemicals and cocktails. (B, C) Statistical data on the ratio of cystic colonies in EFI-based medium with chemical stimulations. (D) Statistical

analysis on the size of colonies in EFI-based medium, supplemented with Acetyl cysteine (Cys), IAFSNT (-VPA) and VIAFSNT. (E) Images of OMP-TdT$^+$ colonies cultured in NWR-based medium with different chemical treatments. (F, G) Statistical data on the ratio of cystic colonies cultured in NWR-based medium with various chemical supplements. (H) Size of colonies in NWR-based medium with treatment of Cys, IAFSNT (-VPA) and VIAFSNT. Statistical difference was determined by one-way ANOVA with Dunnett's multiple comparisons test. Green and black asterisks in (C) and (G) represented the statistical difference compared to the VIAFSNT and untreated (EFI and NWR) group. *$p < 0.05$, **$p < 0.01$, ***$p < 0.001$. Scale bars, 100 μm.

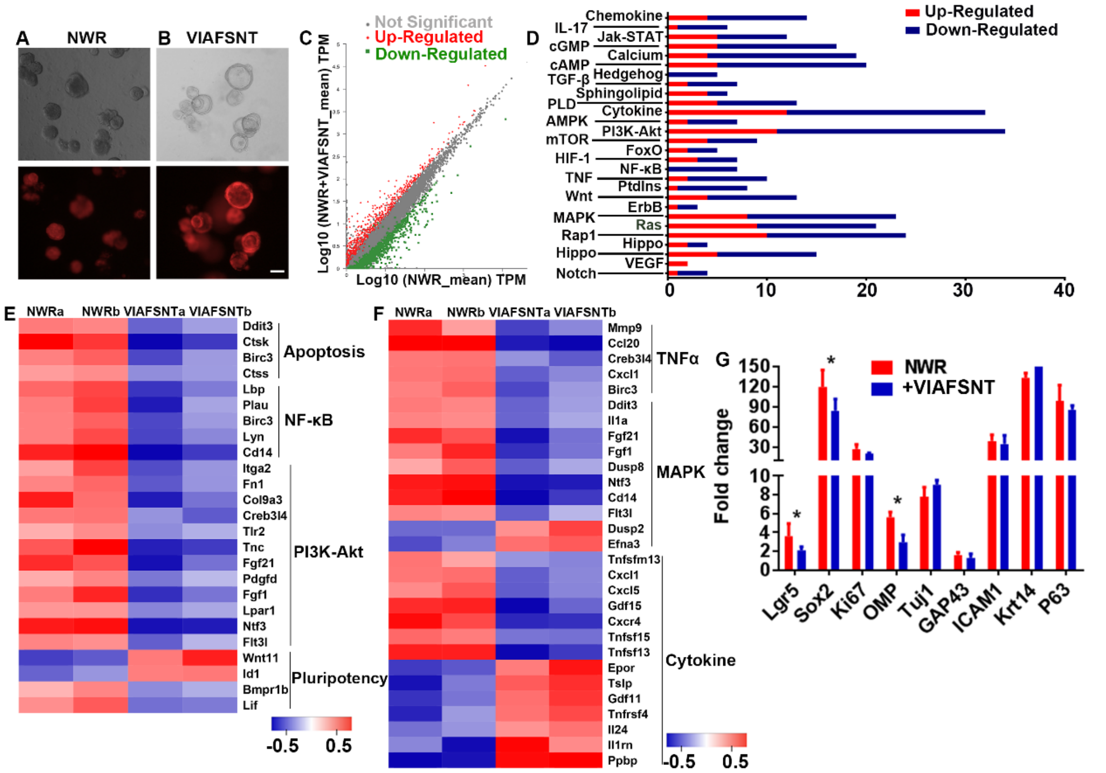

Supplementary Figure 5. Transcriptional alteration in VIAFSNT-treated filled and cystic colonies cultured in NWR-based medium. (A, B) Images of untreated and VIAFSNT-treated OMP-TdT^{+} colonies in NWR-based medium. (C) Scatter plot showed up-regulated, down-regulated and not significantly differentially expressed genes between untreated and VIAFSNT-treated colonies. (D) KEGG enrichment analysis showed that differentially expressed genes between untreated and VIAFSNT-treated colonies participated in multiple signaling pathways. (E) Heatmap of differentially expressed genes participating in apoptosis, NF-κB, PI3K-Akt and pluripotency-associated signaling pathways. (F) Heatmap of significantly up- and down-regulated genes involved in TNFα, MAPK and cytokine receptor interaction pathways between untreated and VIAFSNT-treated colonies cultured in NWR-based medium. (G) Transcriptional fold change of OE cellular biomarkers between untreated and VIAFSNT-treated colonies. Statistical difference was determined by two-way ANOVA with Sidak's multiple comparisons test. *$p < 0.05$. Scale bar, 100 µm.

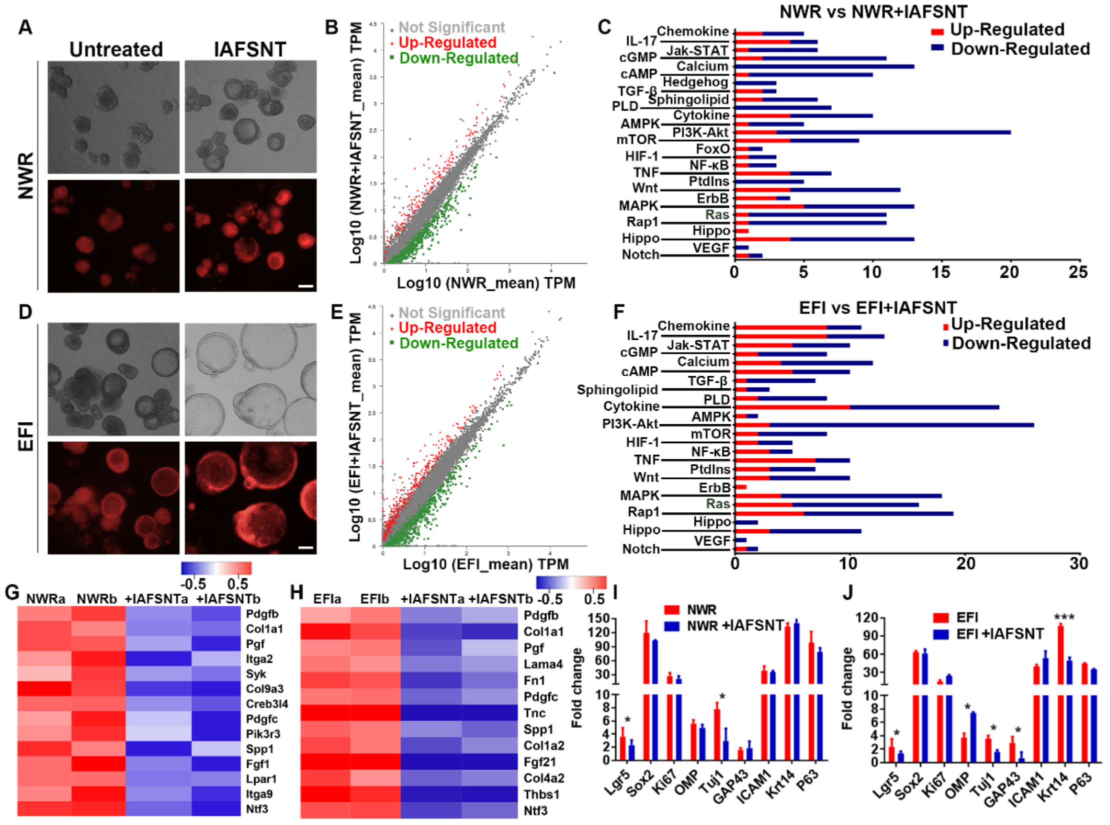

Supplementary Figure 6. Transcriptional difference between untreated and IAFSNT-treated filled and cystic colonies. (A, D) Image of untreated and IAFSNT-treated OMP-TdT^{+} colonies cultured in NWR- and EFI-based media. (B, E) Scatter plot showed differentially expressed genes in untreated and IAFSNT-treated colonies. (C, F) KEGG enrichment analysis showed up- and down-regulated genes involved in multiple signaling pathways in untreated and IAFSNT-treated colonies. (G, H) Heatmaps of down-regulated genes participating in PI3K-Akt signaling pathways in IAFSNT-treated colonies, compared to untreated colonies cultured in NWR- and EFI-based media. (I, J) Transcriptional fold change of OE cellular biomarkers between untreated and IAFSNT-treated colonies. Statistical difference was determined by two-way ANOVA with Sidak's multiple comparisons test. *$p < 0.05$, ***$p < 0.001$. Scale bars, 100 μm.

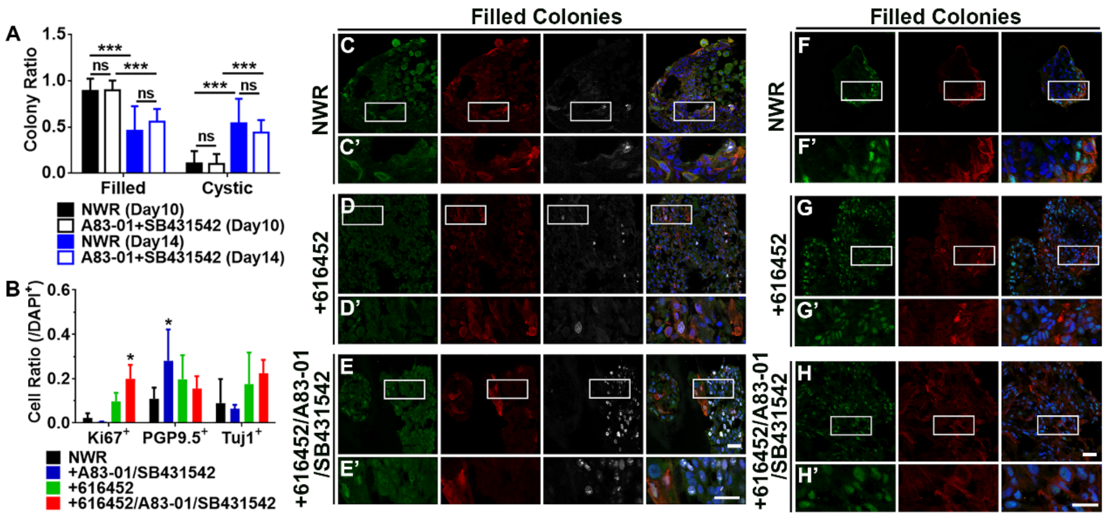

Supplementary Figure 7. Characterization of colonies with filled appearance derived from human olfactory mucosa under different chemical stimulations. (A) Statistical analysis on the ratio of cystic and filled colonies under A83-01/SB431542 treatment on Day 10 and 14 post *in vitro* culture. (B) Statistical analysis on the ratios of Ki67$^+$, PGP9.5$^+$ and Tuj1$^+$ cells in human colonies with treatments of A83-01/SB431542, 616452 and 616452/A83-01/SB431542. (C-H) Immunostaining against OMP, PGP9.5 and Ki67 in untreated colonies (C), colonies treated with 616452 (D) and with 616452/A83-01/SB431542 (E), as well as against Sox2 and Tuj1 in untreated (F), 616452- (G) and 616452/A83-01/SB431542-treated colonies (H) cultured in NWR-based medium. The squared regions in (C-H) were shown as C'-H'. Statistical difference was determined by two-way ANOVA with Tukey's multiple comparisons test in (A) and with Sidak's multiple comparisons test in (B). ns, not significant, *$p < 0.05$, ***$p < 0.001$. Scale bars: 20 μm.

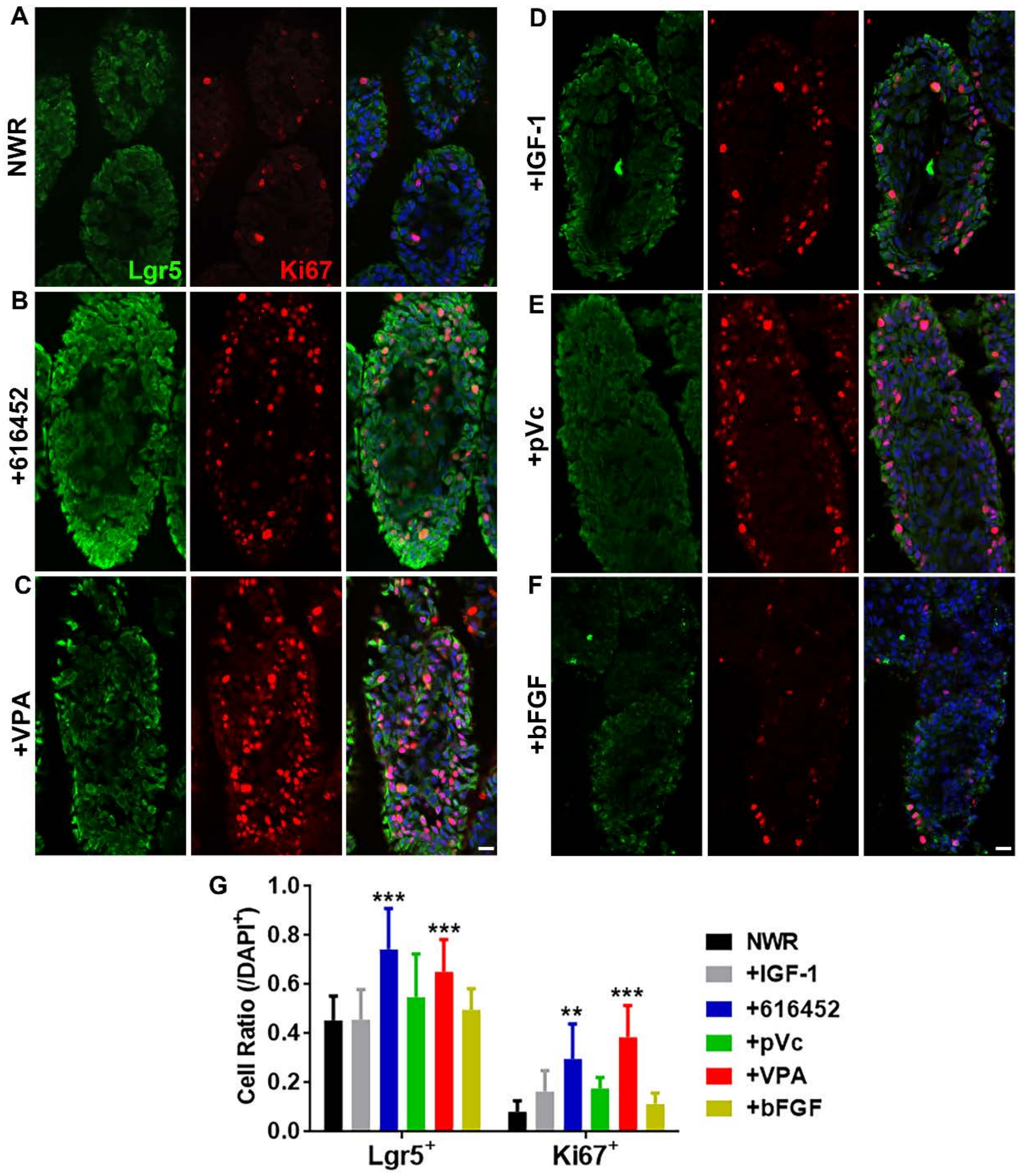

Supplementary Figure 8. Chemicals changed Lgr5 expression and proliferation in colonies derived from human olfactory mucosa. (A-F) Immunostaining against Lgr5 and Ki67 in untreated colonies (A) and colonies with stimulation of 616452 (B), VPA (C), IGF-1 (D), pVc (E) and bFGF (F). (G) Statistical analysis on the ratios of $Lgr5^+$ and $Ki67^+$ cells under various chemical stimulations. The statistical difference was determined by two-way ANOVA with Dunnett's multiple comparisons test. $^{**}p < 0.01$, $^{***}p < 0.001$. Scale bars: 20 μm.
